# Evidence for the importance of land use, site characteristics and vegetation composition for rooting in European Alps

**DOI:** 10.1038/s41598-021-90652-2

**Published:** 2021-05-27

**Authors:** Erich Tasser, Sonja Gamper, Janette Walde, Nikolaus Obojes, Ulrike Tappeiner

**Affiliations:** 1 Institute for Alpine Environment, Eurac Research, Drususallee 1, 39100 Bozen/Bolzano, Italy; 2 Department of Statistics, Faculty of Economics and Statistics, Universität Innsbruck, Universitätsstrasse 15, 6020 Innsbruck, Austria; 3 Department of Ecology, Universität Innsbruck, Sternwartestr. 15, 6020 Innsbruck, Austria

**Keywords:** Biodiversity, Community ecology, Ecosystem ecology

## Abstract

Plant rooting strongly affects most hydrological, biogeochemical and ecological processes in terrestrial ecosystems, as it presents the main pathway for carbon, water and nutrient transfer from soil to the atmosphere and is a key factor in stabilizing the soil layer. Few studies have actually investigated the link between phytosociological and structural vegetation composition and diversity in soil rooting parameters. Our study provides a comprehensive evaluation of plant cover and diversity effects on rooting parameters dependent on different land-use types along a north–south transect in the Eastern Alps. We conducted field studies of root biomass, rooting density and rooting depth for the six main land-use types: intensively and lightly used hay meadows, pastures, arable land, agriculturally unused grasslands and forests. The variation in rooting parameters was explained by different aspects of species and functional richness, species and functional composition, functional traits, abundance of key species and site variables depending on the land-use types. Our results showed that different characteristics of biodiversity explained the variance in root parameters (mass, density and depth) to a high degree (determination coefficient R^2^ values varied between 0.621 and 0.891). All rooting parameters increased with increasing plant species richness, as well as with a higher diversity of plant functional traits. The inclusion of site parameters significantly increased the explained variance, while we could not find evidence for key species and their abundance to provide additional explanatory power. Allowing the effects to vary depending on land-use types turned out to be a necessity supporting the importance of considering land-use types for rooting. The findings indicate that vegetation composition has a clear relationship with rooting parameters across different habitats in the European Alps. As the effect of plant composition differs with respect to the land-use type, rooting can be monitored by land management to achieve the desired benefits. For example, intensified rooting through extensive management decreases erosion risk and increases carbon uptake.

## Introduction

Plant rooting distribution strongly affects most hydrological, biogeochemical and ecological processes in soils and represents one of the main pathways for carbon transfer to the soil^1^ and for water and nutrient uptake by plants^2,3^. Moreover, primary belowground production is often larger than aboveground production, especially in alpine grassland^4^, tundra, tropical/subtropical/temperate arid shrubland and desert ecosystems^5^. Annual carbon and nutrient inputs from roots to the soil often equal or exceed those from leaves^6^.

**Figure 1:**
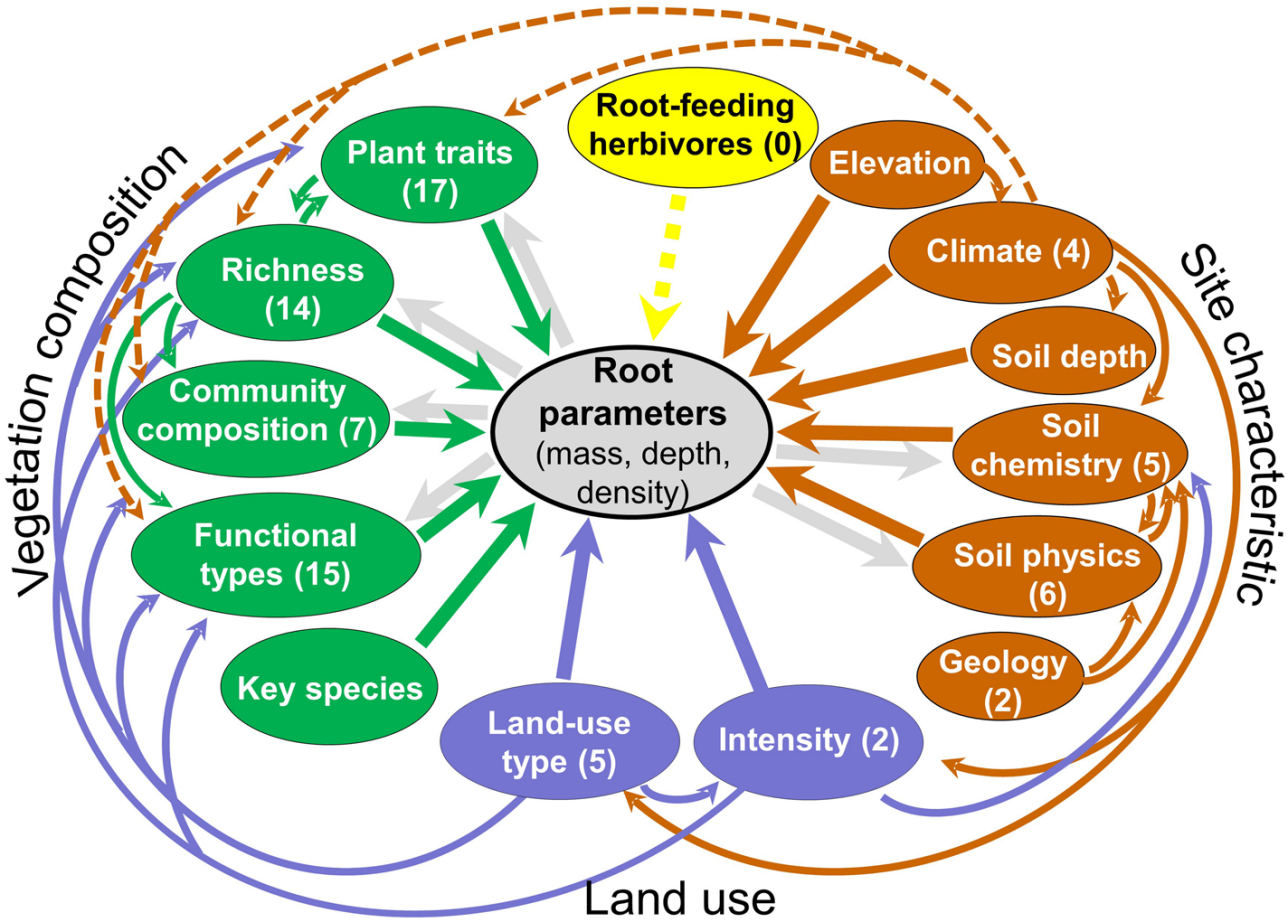
Conceptual framework to analyze the effects of vegetation composition, land-use types and site characteristics on different rooting parameters. The solid arrows between the ovals indicate the relationships considered in this study, whereas the dashed relationships were not considered. Yellow ovals = consumer variables; blue ovals = land-use variables; brown ovals = site and soil variables; and green ovals = vegetation variables. The numbers in the brackets indicate the number of variables used in this study.

Rooting distribution is directly and indirectly affected by many environmental factors (Fig. 1). Broadly, they can be classified into site characteristics (e.g., soil chemistry or climate), vegetation composition (e.g., species richness or community composition), land-use types and root-feeding herbivores. Previous studies have already identified a number of direct effects of site characteristics on rooting^7–10^. Root mass and root length density^11^, as well as root diameter and root penetration depth^12^, were also used as rooting parameters in our study. According to these studies, root morphology and belowground biomass depend on physical soil conditions (soil type, soil depth, organic matter, humus content, water content, and soil aeration) and on chemical and nutrient conditions (pH, P, N, C, Ca, and Mg)^13,14^. Other studies have demonstrated the effects of changing climatic conditions along elevation and latitudinal transects^6,15–17^. The impacts of the abundance of root-feeding insect herbivores^14^ on rooting parameters have also been demonstrated. Furthermore, few studies have investigated the direct link between rooting parameters and vegetation^18–22^.

The explanatory vegetation variables frequently used are plant cover, species/functional richness, plant traits and functional composition^23^. Findings have either been obtained with observational studies comparing biodiversity and rooting parameters in different ecosystems or with manipulative biodiversity experiments using mainly single ecosystems (for a review see^24^). In most cases, a positive relationship was found between functional diversity (e.g., the diversity of functional traits) and functional composition (e.g., the presence of a certain functional group) and average belowground biomass production^22,25,26^. Authors^12,27^ have underlined the importance of key species, increasing the diversity of root types and root functions. Evidence is in the literature of significant direct effects of land-use types on rooting^18,28^. Plowing the soil for cultivation of agricultural crops drastically reduces the amount of belowground biomass and changes the vertical root distribution^28^.

Finally, feedback effects between rooting and individual environmental and vegetation variables are known. For example, rooting densities and vertical distribution patterns affect soil properties, such as aggregate stability, water content and soil aeration, or control the establishment of specific plant compositions^2,29^. These interdependencies need to be taken into account when interpreting the estimates obtained from regression models. Therefore, the estimates are meant to provide *associations* and correlation measures but do not yet allow for a strict, mono-directional causal interpretation.

In addition to the direct effects, there is evidence in the literature for indirect effects influencing rooting (see Fig. 1). A large number of these indirect effects are triggered by land-use types and intensity. Fertilization, mowing and grazing affect soil chemistry and, consequently, soil rooting^18^, while the use of heavy agricultural machines^30^ leads to soil compaction. Land use has even more severe effects on vegetation composition, as certain land-use types and intensities favor specific functional groups and eliminate others^31–33^. On arable land, only certain crop plants and annual weeds occur; most other species are repressed physically or chemically^34^. Abandonment of land use leads to the (long-term) establishment of different (secondary) climax plant communities.

In addition to land use, climate may also trigger indirect effects on vegetation and soil development. Seasonal dry periods favor higher rooting depths to provide access to resources in deeper soil layers during drought^19^. Climate controls the establishment of characteristic plant species, functional groups and plant traits in abandoned areas^35^. Numerous studies have documented the indirect effects of soil texture on rooting parameters due to its influence on water and nutrient availability (for a review see^20,36^). Furthermore, intraspecific or interspecific competition between species leads to changes in rooting parameters^19,37^.

The role of individual influencing variables directly or indirectly in this complex system is largely unclear, in particular the role of vegetation and the effects of biodiversity changes on rooting parameters. It is generally known that plant community composition and diversity have significant effects on ecosystem functions^25,38,39^. Higher biodiversity favors biomass production^39,40^; in many cases, this has positive effects on soil nutrient foraging and on decomposition (but not always). Plant diversity is hypothesized to increase soil stability and to improve soil properties, such as aggregate stability, infiltration capacity and soil bulk density, by increasing the diversity of root types^41,42^ and by better preserving ecosystem integrity under severe stress^43^. Surprisingly, there was no evidence that biodiversity is related to soil carbon storage^26^. Most of these relationships were derived from single-ecosystem studies and were obtained experimentally under controlled conditions. In these studies, biodiversity was manipulated, and the responses of ecosystem functions were assessed. However, the extent to which these results actually reflect processes in the real world has been the subject of scientific debate for some time, and a final decision has not yet been reached^26^. It is clear that these results must also be confirmed with field studies. With our study, we contribute to closing this gap in scientific knowledge.

According to evolutionary principles (cf.^44^), we assume that vegetation adapts to optimize the use of its local environment. Tello-García and colleagues observed a massive change in above- and belowground phytomass in a climate experiment on mountain grasslands^45^. The authors attributed this change more to a change in the vegetation composition and less to changes in the morphology of individual species. Similar results have been documented by other authors in long-term studies^46^. We conclude that changes in land use or site conditions do not primarily lead to changes in rooting morphology but rather to shifts in vegetation composition^3,23^. We hypothesize that vegetation changes are the main reason for significant changes in above- and belowground biomass.

In this study, we therefore determined the associations of vegetation composition (plant cover, species/functional richness, plant traits and functional composition) on rooting. We compared ecosystems (communities) with different vegetation compositions and diversities in this study across ecosystems using the most important site and land-use variables. We used principal component analysis (PCA) and regression analysis to investigate a broad cross-section of land-use types (arable land, intensively and lightly used meadows, pastures, agriculturally unused grasslands and forests) along a north–south transect in the Eastern Alps. Our specific hypotheses were the following:

- Vegetation composition (Fig. 1) comprises the main determining factors for root mass, root length, and rooting depth.
- Within the vegetation composition, functional types and plant traits influence rooting the most, less so species richness.
- No effect by a single key plant species on rooting parameters is present.

Our hypotheses thus concerned rooting across ecosystems, i.e. we investigated ecosystem-wide associations of vegetation composition, site characteristics and land use with rooting.

## Material and methods

### Study sites

**Figure 2:**
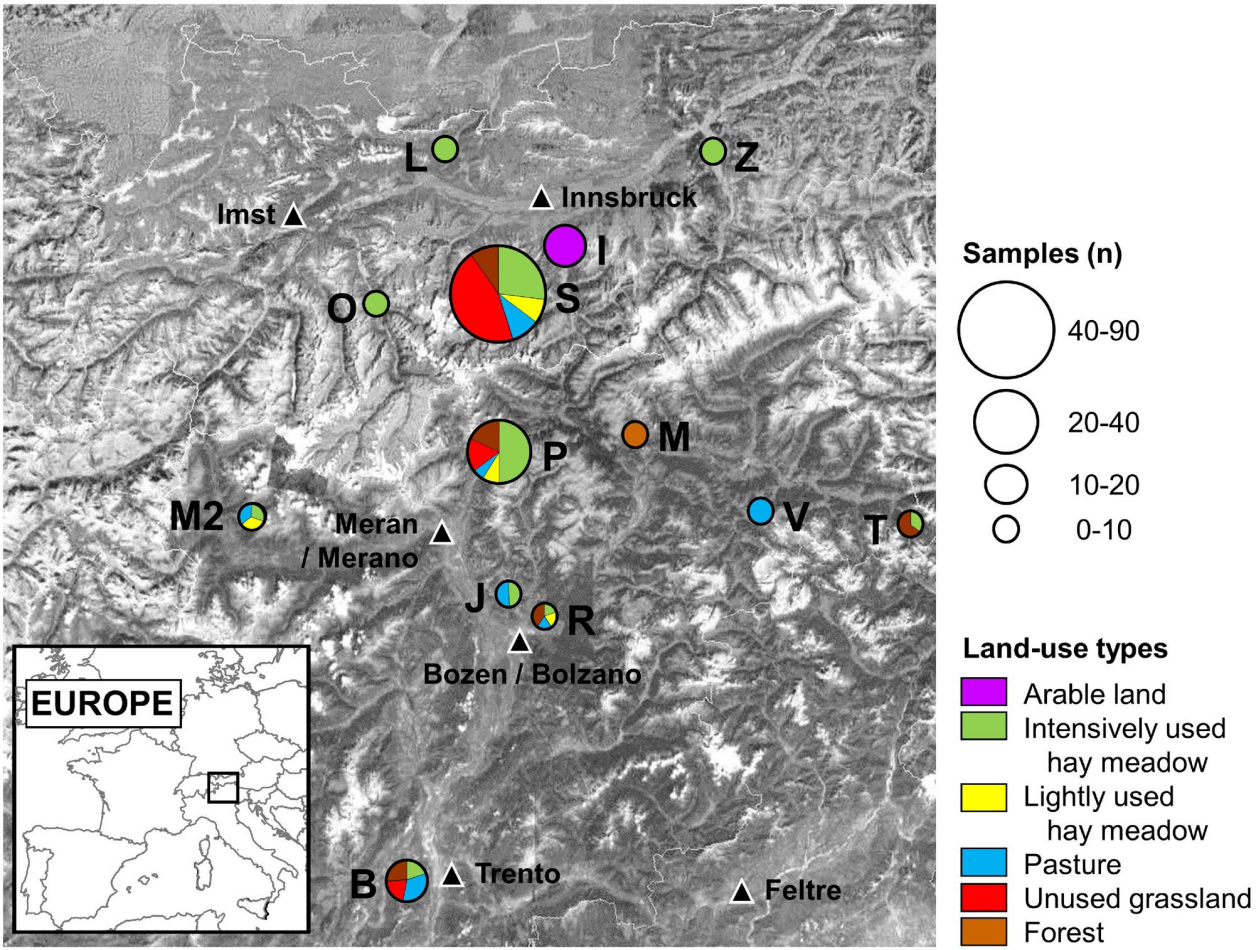
Site, sample number and analyzed land-use types in the Eastern Alps. Study sites: B = Monte Bondone; I = Igls/Patsch; J = Jenesien; L = Leutasch; M = Mühlbach; M2 = Matsch; O = Ötz Valley; P = Passeier Valley; R = Ritten; S = Stubai Valley; T = Toblach; V = St. Vigil; and Z = Ziller Valley. The map was created using ArcGIS 10.2.2 (ESRI Inc.) and edited in Microsoft PowerPoint 365 MSO (Map data: Esri, DigitalGlobe, GeoEye, Earthstar Geographics, CNES/Airbus DS, USDA, USGS, AEX, Getmapping, Aerogrid, IGN, IGP, swisstopo, and the GIS User Community).

To obtain a cross-section of land-use types through the Eastern Alps (Fig. 2), rooting samples were taken from Tyrol (Austria) and from South Tyrol and northern Trentino (both in Italy), which include two climatic regions—the central European climatic region in the northern part and the sub-Mediterranean climatic region in the southern part of the research area^47^. The average annual precipitation at the 13 study sites ranges from 400 to 2000 mm, with maximum rainfall observed from June to July^47^. Mean annual temperature ranges from 0 °C to 9 °C. Additional climatic variability was added by sampling at elevations from 650 to 2680 m a.s.l. The bedrock in the research area is composed of calcareous sedimentary rock in the northern and southern regions and of crystalline rock in the main chain of the Alps, sometimes with superimposed calcareous isles: Stubai Valley (North Tyrol) is geologically dominated by silicate with transitions to limestone; Ötz Valley, Ziller Valley and Igls/Patsch (all North Tyrol), Passeier Valley, Mühlbach, Matsch, Ritten and Jenesien (South Tyrol) are geologically dominated by silicate; and Leutasch (North Tyrol), St. Vigil and Toblach (both South Tyrol) and Monte Bondone (near Trento) are geologically dominated by limestone. The pH of the topsoil (0–10 cm), which ranges from 3.7 to 7.8^32^, is determined by bedrock and land use^48^. For more details on the study region, see Supplementary Appendix S1.

To be representative, the most widespread vegetation communities in the 13 study sites for all land-use types (arable land, intensively used hay meadow, lightly managed hay meadow, pasture, agriculturally unused grasslands, and forest) were analyzed (Supplementary Appendix S2). Overall, a total of 171 soil samples were taken, with 15 samples from arable land, 56 samples from intensively used hay meadows, 15 samples from extensively managed hay meadows, 16 samples from lightly stocked pastures, 32 samples from agriculturally unused grasslands, and 37 samples from forests. Meadows that are mown and fertilized with slurry and/or manure at least twice a year were defined as intensively used hay meadows. An extensively managed hay meadow was not fertilized and mown only once a year. Pastures were extensively grazed by cattle and/or sheep (annual average stocking intensity: 0.15–0.4 livestock units (LU) ha^−1^ year^−1^) but not mown. As arable land, we defined different crops typical for the region, especially maize and bread cereal crops, as well as vegetables and potatoes. Agriculturally unused grasslands included all grassland areas that were abandoned for at least five years or have never been used for agriculture, such as alpine grasslands. Finally, all permanent deciduous, coniferous or mixed forests were combined into the forest land-use type (thus, no energy forests).

### Data collection and analysis

Vegetation and site variables depending on land-use types were used to explain the rooting parameters. As Fig. 1 shows, dependencies between explanatory variables and rooting parameters are not always strictly unidirectional. For example, vegetation composition influences rooting; however, rooting patterns can also influence vegetation composition. We considered as many different dependencies as possible in the applied methods and interpreted discovered statistically significant relationships as associations rather than causal (unidirectional) impacts.

#### Rooting parameters: root mass, root length and rooting depth

Overall, 171 rooting samples (Appendices S1 and S2) were taken between 1994 and 2017 in the field with core samplers of 6.8–7.7 cm diameter and a maximum core depth of 70 cm. Before coring, the vegetation was characterized with the standard phytosociological method of Braun-Blanquet^49^ to directly connect rooting and vegetation characteristics. The size of the vegetation survey areas was determined by the minimal area of a plant community as the area with 90% or more of all plant species within this ecosystem. The survey area ranged between 1 m × 1 m in homogenous meadows and 10 m × 10 m in forests. Even though we chose the rooting survey areas to be homogeneous regarding vegetation composition, it was possible that the rooting measured in the soil cores was affected by species other than those located above the core area due to large heterogeneity within plant communities^50^. Nevertheless, this error should be negligible.

As the data for this analysis were derived from a collection of rooting analyses from different research projects in the past 20 years using the same methodological approach, the number of samples per land-use type and per site was unbalanced (Supplementary Appendix S2). For example, some land-use types were represented only at one site (e.g., all agriculturally unused areas were at site I), while others were represented at three or even more than 10 sites. In addition, the number of samples within each land-use type was also unbalanced: 15 samples for arable land, 32 for agriculturally unused grasslands or 56 samples for intensively used hay meadows. The original data collection included the most common and important plant communities in the project areas except for arable land. Thus, the rooting of the most common crops (maize: n = 3; barley: 3; oat: 3; wheat: 3; and vegetables: 3) was analyzed near Innsbruck in an area specially selected for this purpose.

In the laboratory, the soil cores were split into the O-horizon (if present) and mineral soil layers of various thicknesses (0–3 cm, 3–8 cm, 8–13 cm, 13–23 cm, 23–38 cm, 38–53 cm, and > 53 cm). Root extraction was performed manually with the roots cleared of soil in sieving cascades under running water^51^. Afterwards, the roots were sorted into three size categories^18^: (1) very fine roots (diameter between 0 and 1 mm); (2) fine roots (diameter between 1 and 5 mm); and (3) coarse roots (diameter between 5 and 20 mm). Roots of woody species with a diameter larger than 20 mm were not taken into account, as the distribution and diameter of coarse roots (especially trees) in the soil vary greatly spatially; therefore, a single survey cannot be representative of the rooting of an ecosystem^50,52^. The reason for this classification was due to the different functions of the classes. Very fine roots have a dominant role in the uptake of water and nutrients and may be the main source of stabilized carbon input to soil^1^. Fine roots are mainly responsible for the transport, anchoring and storage of carbohydrates and are also able to take up water. Coarse roots are important for water transfer and the stabilization of plants. To account for the different specific root lengths (SRLs) of very fine and coarse roots from herbaceous and woody species^29^, we classified the single samples according to the cover of herbaceous and woody species from the phytosociological surveys into pure grassland samples, mixed grassland samples (dominance of woody species: < 50%) and dwarf shrub-rich or tree-rich samples (dominance of woody species: > 50%)^18^. The conversion of root mass to rooting length was carried out using previously published Eqs. ^19^ (Table 1). Finally, the maximum depth (*RD*_90%_), above which 90% of the total root mass was found, was calculated for each root sample using the equation:

1 $$RD_{90\% } = RM_{tot} \frac{{\arctan \left( {\frac{{RM_{90\% } }}{{RM_{tot} }}} \right)}}{{m_{\max } }},$$

where *RM*_90%_ is 90% of the total root mass (kg m^-2^) and *m*_max_ is the maximum slope of the saturation curve. In the same way, the depths above which 50% (*RD*_50%_) and 95% (*RD*_95%_) of the total root mass occurred were calculated. In forests and in dwarf shrub-rich communities, the rooting depths and distributions could be biased by the fact that the sampling depth was very shallow, which could lead to underestimating the 50%, 90% and 95% rooting depths^53^. In grassland ecosystems, croplands and in dwarf shrub rich communities, however, the 70 cm sampling depth is sufficient because most roots are within the top 30 cm^18^.

**Table 1:** Linear functions to calculate the root length on the basis of root dry weight for different vegetation communities: grassland communities (G), mixed grassland communities (M), and dwarf shrub-rich or tree-rich vegetation communities (W). y = root length (mm m^-2^) and x = root dry mass (g m^-2^).

Root diameter types	Linear function
Root diameter: 0–1 mm (G)	y = 884.3 + 103,282.0*x
Root diameter: 0–1 mm (M)	y = 118.6 + 56,453.0*x
Root diameter: 0–1 mm (W)	y = 196.2 + 26,518.6*x
Root diameter: 1–5 mm (G/M/W)	y = 89.6 + 693.7*x
Root diameter: > 5 mm (G/M)	y = 28.2 + 123.3*x
Root diameter: > 5 mm (W)	y = − 39.7 + 100.12*x

#### Environmental variables

For every root sample, we collected 79 potential impact variables on rooting, including 19 site variables, six land-use variables and 53 vegetation variables (see Table 2 and Appendices S1, S3 and S4).

**Table 2:** Groups of variables used to explain rooting parameters, including information on the type (V, vegetation variable; S, site variable; and LU, land-use variable), the number of variables of each group (no.) and examples (for details, see Appendices S1, S3 and S4).

Group of variables	Type	no	Examples
*Richness*	V	14	Total vascular plant species number, species numbers of different taxonomical groups and functional types
*Community composition*	V	7	Total vegetation cover, mean species cover, plant cover variance or dominance of individual species, Shannon–Wiener and Evenness index of species abundance
*Cover of functional types*	V	15	Relative abundance of functional types (lichen, mosses, ferns, true grasses/Poaceae, rush grasses/Juncaceae, sedges/Cyperaceae, legumes, non-legume forbs, dwarf shrubs, trees), Shannon–Wiener and Evenness index of functional types
*Community-level traits*	V	17	Traits (rooting density, rooting depth, leaf area, plant height), Shannon–Wiener and Evenness index of functional traits
Site characteristic	S	19	Climate (temperature and precipitation), elevation, soil depth, soil physical and chemical properties, geological basis
Land-use type and intensity	LU	6	Arable land, intensively used hay meadow, lightly managed hay meadow, pasture, agriculturally unused grasslands, forest

##### Vegetation variables

In total, 53 vegetation variables were collected and divided a priori into four groups (Table 2, Supplementary Appendix S3). Variables included in the *richness* group were ‘number of plant species’, ‘number of taxonomic groups’ and ‘functional types’ (after^38^). All variables that displayed information on the mean species cover, plant cover variance or dominance of species, the Shannon–Wiener and Evenness indices (both after^54^) and the total vegetation cover were allocated to the *community composition* group. We calculated the Shannon–Wiener and Evenness indices^54^ for species composition, functional types and functional traits.

The *cover of functional types* group included variables that provide information on the abundance, dominance and composition of single plant functional types (see Supplementary Appendix S3). Finally, the *community-level trait* group (see Supplementary Appendix S3) contained leaf, plant height and root traits (effect traits in sensu^55^) used to assess the relative effects of aboveground and root trait turnover at the community level. They were calculated for each sample using trait values taken from the literature and the measured abundance of each species within the single community (i.e., community weighted mean^56^). We used mean root density and main rooting depth for the single species^57–59^. The rooting density of the species was classified into sparse, medium dense, dense, and very dense roots^59^. The mean leaf size and plant height of the species (sources:^60,61^; http://www.floraweb.de/; own observations) were classified according to the following thresholds. Plant height was divided into small (mean plant height < 20 cm), medium (20–40 cm), large (40–90 cm) and very large (> 90 cm) species. Leaf size was classified as small (mean leaf area < 10 cm^2^), medium (10–70 cm^2^) and large-leaved (> 70 cm^2^) species. In accordance with other authors^62,63^, most plant species showed clear allometric allocation trends between leaves, stems and root biomass for different groups of plant species. In particular, a trend towards a decreased root mass fraction with plant size was detected.

##### Site characteristics

Important meteorological parameters were measured at eight study sites at a distance of < 150 m from the rooting samples using different microclimate stations. For the five sites without site-specific climate measurements, we used data from standard meteorological stations of the regional or national Weather Service departments at a distance of < 5 km and at approximately the same elevation.

For detailed soil characterization, soil profiles were investigated directly at the sample site or at a representative site with the same land-use type and identical plant communities in the immediate vicinity (< 50 m distance). Undisturbed soil samples of 250 cm^3^, as well as disturbed soil samples (~ 2 kg), were collected from the main root horizon for subsequent physical and chemical analyses. The disturbed soil samples were air dried and passed through a 2 mm sieve. For all soil samples, we analyzed pore size distribution, soil bulk density, soil particle density, total soil porosity, soil texture, and soil organic C and pH (for technical details and exact descriptions of the employed methods, see Supplementary Appendix S13).

Furthermore, we calculated mean Ellenberg's indicator values (EIV^64^) for temperature (T), moisture (F), soil reaction (R) and soil productivity or fertility (N) for all study sites. EIVs are bioindicators for ecological requirements in Central Europe of a single plant species in a competitive relationship. We weighted the species-specific EIVs with the cover of each species from the vegetation relevées after Braun-Blanquet^49^ to create a community-weighted mean for each site.

##### Land-use types

Past and present management practices at the study sites were recorded by interviewing landowners. All landowners were asked to specify the actual type (arable land, hay meadow, pasture, agriculturally unused grasslands, or forest) and intensity of use (intensively or extensively managed) or the number of years since abandonment.

##### Key species

The idiosyncratic effects of particular species must be accounted for^65^. These are effects that cannot be satisfactorily explained by the weighted mean or distribution of functional composition or traits but rather by the abundance of particular plant species^12,27^. Therefore, these key species are not specific to a functional type of plant; they may belong to grasses, forbs, shrubs or trees. For this work, we could not or did not want to define key species a priori, but we considered all species with a presence > 1% and a mean plant cover > 1% as potential key species. In sum, we identified 29 potential key species. Our aim was to determine whether one of these potential key species, in addition to the vegetation and site variables, has additional explanatory power for the rooting parameters and thus actually becomes a key species.

### Statistical analyses

As the number of vegetation variables (Supplementary Appendix S3) and site variables (Supplementary Appendix S4) were too many with respect to our sample size (n = 171), we used two principal component analyses (PCAs) to summarize these two sets of variables into components. For each PCA, components were uncorrelated with each other, but variables were highly correlated within the corresponding component. Therefore, each component comprised the common information of the variables correlated with this component. Variables not correlating with any components were included in the further analyses as original variables. In this way, the number of variables used in the subsequent regression analysis could be significantly reduced.

In order to operationalize and test hypothesis 1, separate regressions for each rooting parameter were computed. The estimated standardized regression coefficient with its standard error represents whether the component/variable has a statistically significant partial correlation with the corresponding rooting parameter. To allow for different associations dependent on the land-use type, a separate coefficient for each land-use type was applied (see Fig. 1). The remaining indirect effects of site variables on rooting using vegetation components/variables (Fig. 1, dashed arrows) were not considered (cf. Supplementary Appendix S13). To summarize the associations of the independent components/variables on each rooting characteristic, we counted whether a components/variables was statistically significant within the different rooting characteristics (root mass, root length, rooting depth). The ratio of significant estimates of the vegetation composition components/variables to the number of all estimates of these components/variables was used as a measure of vegetation composition importance. The same procedure was applied to the site characteristics. To provide a measure of the importance of the land-use types, we calculated the ratio of the number of times the estimates for each components/variables were different with respect to land-use type within a rooting characteristic versus all possibilities. Investigating these importance measures provides an answer to the relevance of the vegetation composition compared to site characteristics and land use. To elaborate the importance of the vegetation composition in more detail (hypothesis 2), the ratio of significant estimates of each vegetation group to the number of all estimates of this vegetation group was used as a measure of its importance.

The regressions underlying the computation of the importance measure (used for hypothesis 1 and 2) required many coefficients (number of components/variables multiplied by the number of land-use types). Unfortunately, we did not have enough samples for each land-use type compared to the number of used components/variables. Therefore, we chose a two-step procedure. For the land-use forest types, intensively used hay meadows and agriculturally unused grasslands, the sample sizes sufficed, and the regression with all components/variables was computed. From these calculations, we obtained the statistically significant components/variables (inductive part of the two-step procedure).

Only the significant components/variables from the inductive part were used in the second step for the remaining land-use types (arable land, lightly used hay meadows and pastures). The components/variables with a p-value < 0.15 were considered in the second step to investigate potential relationships with rooting parameters for the remaining land-use types. The second step was therefore an explorative approach but nevertheless gave a first insight into possible important components/variables facing a restrictive sample size.

For all regression analyses, we checked the model assumptions. No severe multicollinearity between the explanatory components/variables was present (variance inflation factor (VIF) < 4). Using residual diagnostics residuals were symmetrically distributed and showed no severe heteroscedasticity. Nevertheless, we used bootstrapped standard errors in our analyses (bootstrap samples 1000). Cook's distance was checked for possible outliers. We examined in detail the samples with Cook’s distance values that were substantially larger than the rest and looked at the relative changes in the estimates when dropping these samples^66^. Very few samples (< 1.8% per model) had to be excluded from the regression analysis, as the relative changes of the estimates (> 25%) indicated highly influential single observations. For all models, the F-tests indicated the necessity of allowing different level and slope coefficients with respect to land-use types (p < 0.05, for details, cf. Supplementary Appendix S5).

To check hypothesis 3 key species for rooting parameters were identified. A PCA was computed with the abundances of all potential key species (presence > 1% and mean plant cover > 1%). We investigated whether all these species were summarized into PCA components, i.e., into species groups with similar habitat requirements. Species not included in any component were treated as their own component (however, in our study, all species were included in a component). The multiple correlation coefficient (R^2^) of each component with the vegetation and site components/variables was computed. A high R^2^ denotes that the information of the key species is covered by the vegetation and site variables.

All technical details and further detailed descriptions of the methods can be found in Supplementary Appendix S13. Statistical analyses were conducted with Stata/MP 13.1 for Windows.

## Results

The 53 explanatory vegetation variables were summarized into 12 vegetation components, each with an eigenvalue larger than 1, using PCA. The proportion of the total variance of the vegetation variables explained was 84% (see Supplementary Appendix S3). The first component (20% of the variance) consisted mainly of variables quantifying the community composition (Evenness and Shannon–Wiener indices) of vegetation types. The second component (11%) comprised different variables expressing the richness of forb and grass species; the third component (8%) included only information on the cover of all cryptogams. Components 4 (7%) and 5 (6%) were dominated by different variables describing the cover of trees or that of lichens only. Components 6 (6%) and 7 (4%) contained different indices describing the cover of functional types (dominance of forbs and sedges). Components 8 (6%), 9 (6%) and 13 (3%) included information describing the community-level traits of aboveground biomass (total plant cover and dominance of small- and large-leaved species). Components 11 (6%) and 12 (3%) referred to the community-level traits of the rooting system (cover of species with medium and very dense rooting density). Finally, the cover of Juncaceae and the number of dwarf shrub species did not load on any of the components. They were therefore included as additional independent variables in the analyses.

Accordingly, the site variables were summarized via PCA. From the original 19 site variables, five components were extracted, which explained 78% of the total variance (Supplementary Appendix S4). Component 1 (28%) summarized high elevation growing conditions, while component 2 (18%) described favorable growth conditions. Component 3 (13%) referred to silty (as opposed to sandy) soils, and component 4 (11%) described calcareous soils with low clay content. Component 5 (8%) included information about the plant-available water in the soil. The soil depth did not load on any of the components and was therefore used as an original variable in the following analyses.

All components and variables (not loading on any of the components) were used to analyze the rooting parameters via regression models.

### Effects on root mass

**Figure 3:**
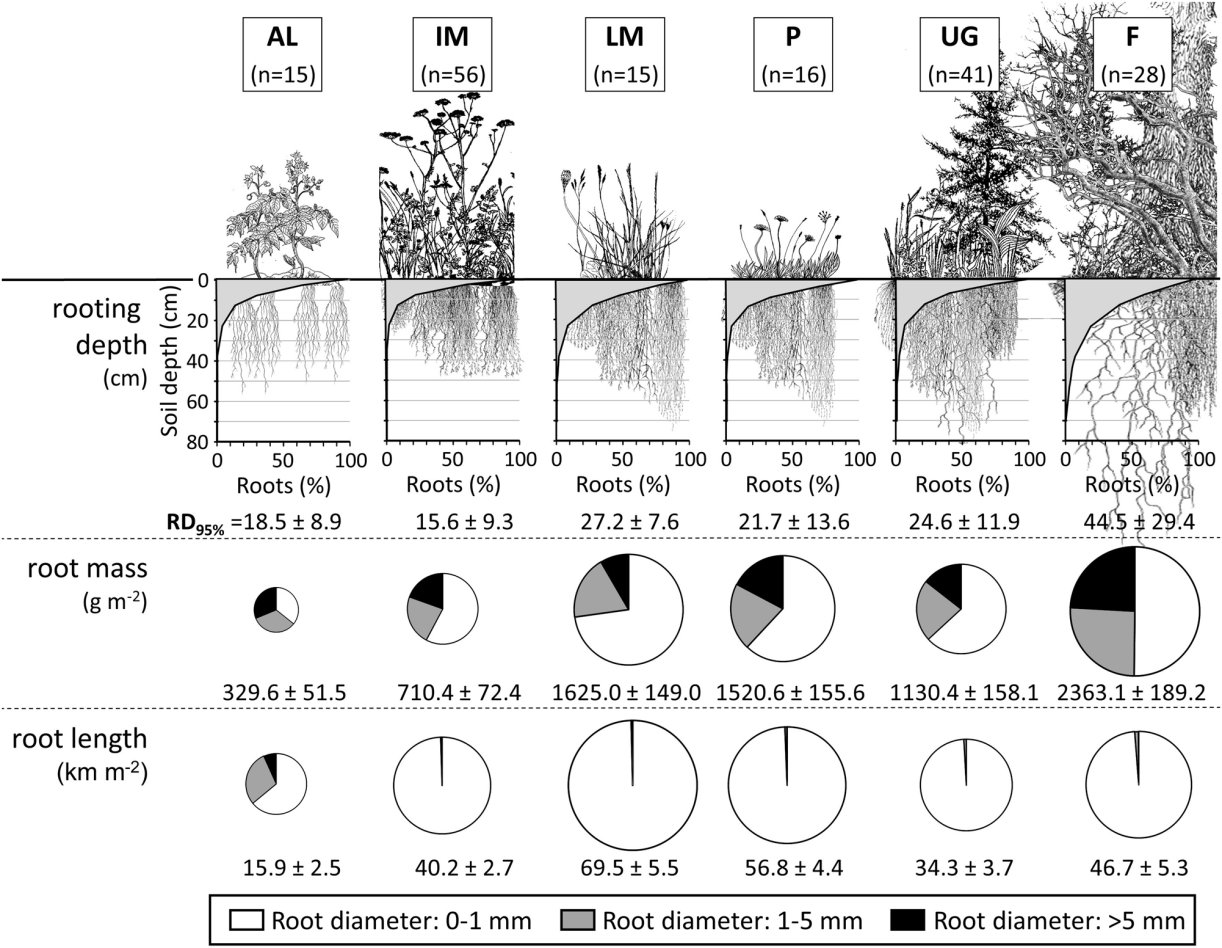
The rooting parameters (root mass, root length, and rooting depth) dependent on different land-use types (AL = arable land, IM = intensively used hay meadow, LM = lightly managed hay meadow, P = pasture, UG = agriculturally unused grasslands, and F = forest). Means and standard errors are given for RD_95%_ (depths above which 95% of the total root mass occurred), total root mass and total root length.

Total root mass as a sum over the entire depth of the soil profile (maximum analyzed soil depth = 70 cm) differed strongly between the analyzed land-use types (Fig. 3). The highest root masses were found in forests and lightly managed hay meadows, and the lowest were found in arable land. In general, the finest roots (0–1 mm in diameter) accounted for the largest share of root mass, ranging from 35% in arable land to 73% in lightly used hay meadow. Coarse roots (5–20 mm in diameter) were mainly found in forests and arable land. A reduction in land-use intensity from intensively used hay meadows to lightly used hay meadows increased root mass.

**Figure 4:**
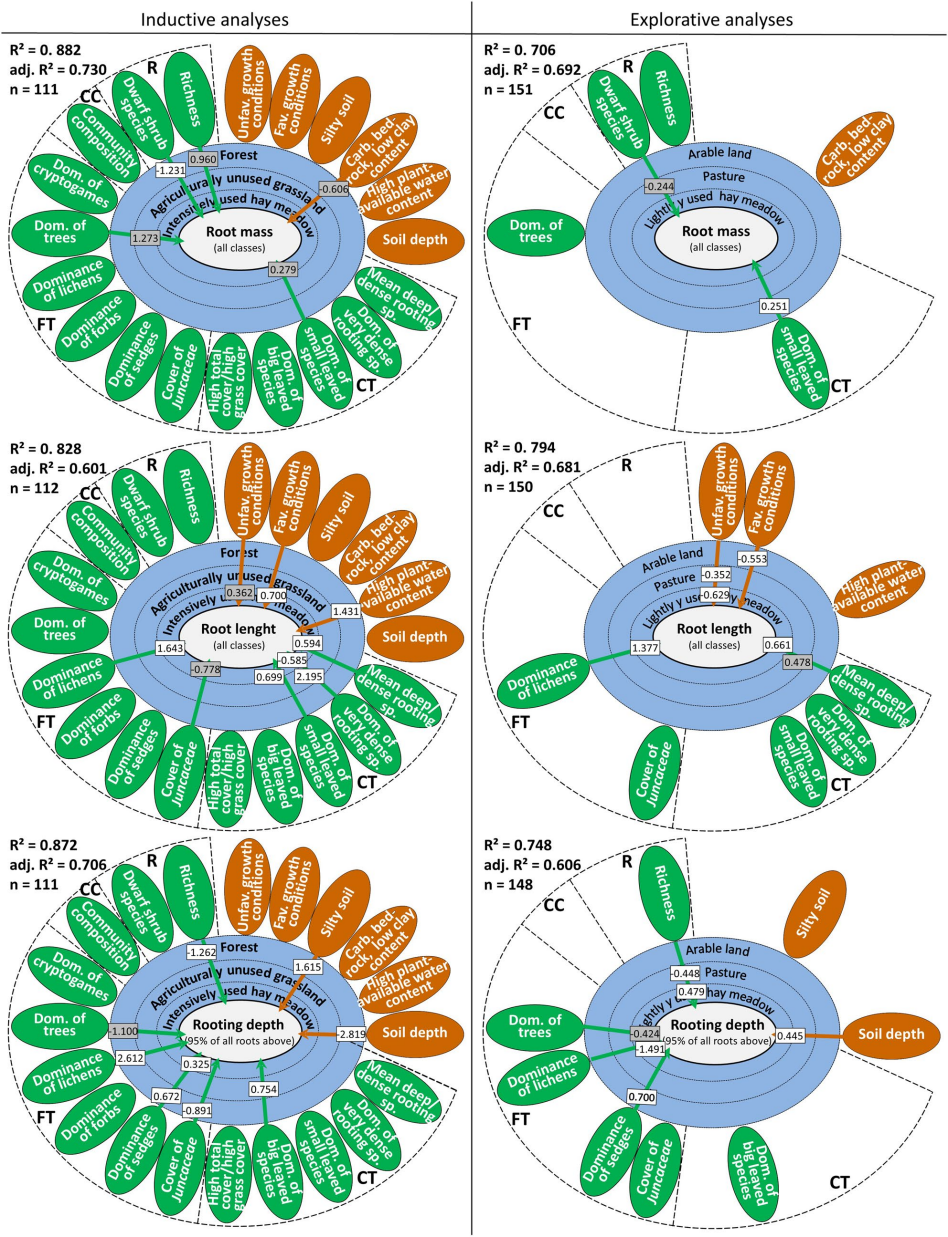
Connection between vegetation composition (green ovals), site characteristics (brown ovals), land-use type (blue rings) and total root mass, total root length and rooting depth (center) showing estimated standardized coefficients for all significant variables (p < 0.05; white rectangles) and near significant variables (p < 0.1, gray rectangles). The standardized coefficients are given in rectangles for each land-use type (i.e., three dashed ovals in the middle) that exerts a significant influence in combination with the corresponding environmental factor. Additionally, R^2^ values, adjusted R^2^ values and sample size (n) are provided for each model. The figure is divided into two panels to clearly indicate step 1 (left panel) of the approach (i.e., the inductive analyses) and step 2 (right panel). In step 2 (cf. also statistical analyses section), only the significant components/variables of step 1 were further used due to sample size limitations, and therefore, these findings have a more explorative character. Detailed results of each single root class are shown in Appendices S6–S11. All diversity components and variables were assigned to one of the diversity indicators groups (R = *richness*, CC = *Community composition*, FT = *cover of functional types*, and CT = *community-level traits*).

**Figure 5:**
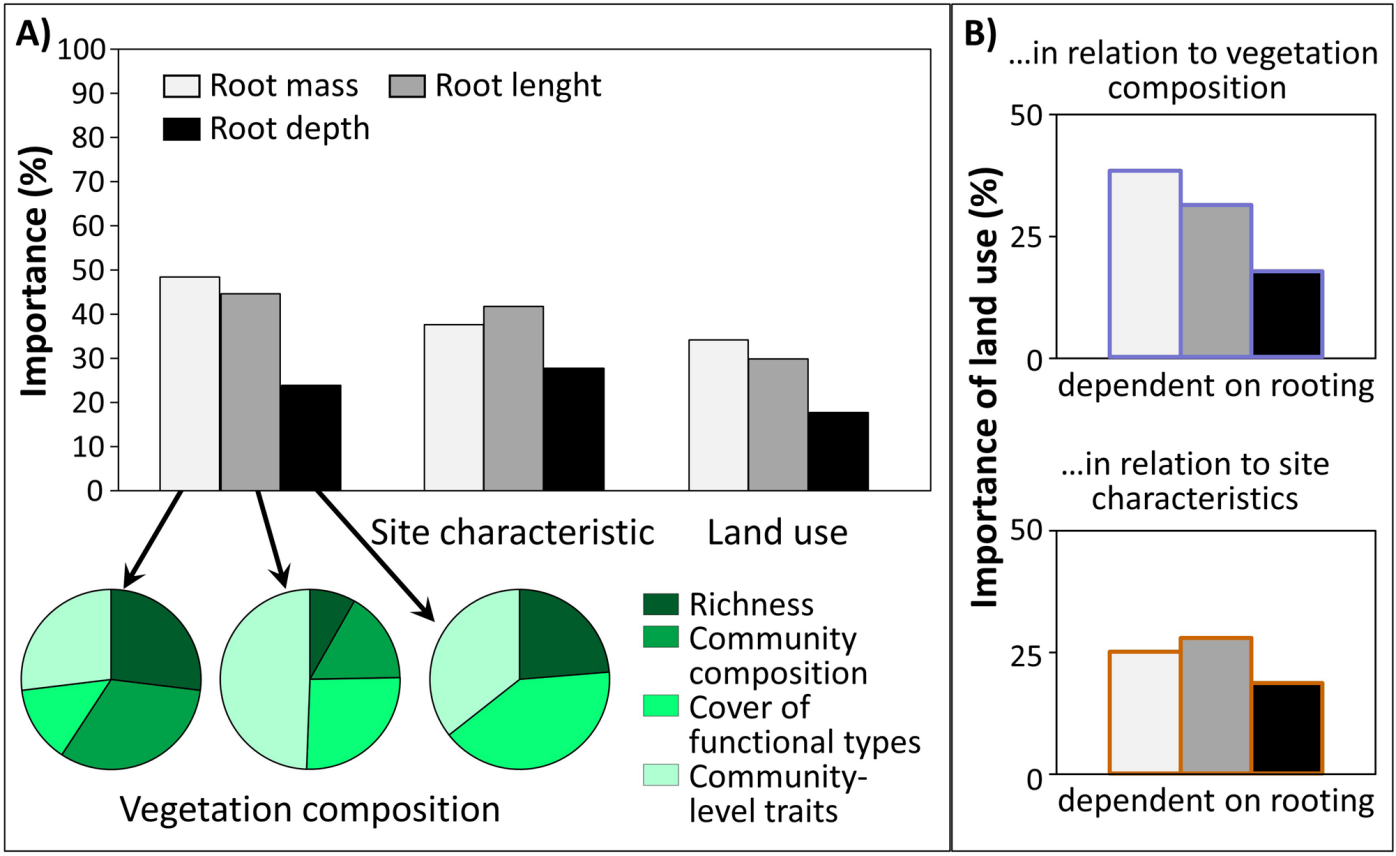
(**A**) Importance (%) of vegetation composition and site characteristic as well as both in relation to land use is shown dependent on rooting (root mass, root length, and rooting depth). The importance of each vegetation composition group (*richness*, *community composition*, *cover of functional types*, and *community-level traits*) is provided. (**B**) Importance (%) of land use in relation to vegetation composition or site characteristics dependent on rooting is plotted.

Root mass was explained to a high extent by vegetation and site variables for different land-use types (Figs. 4 and 5; Supplementary Appendix S6): 88.2% of the variance in total root mass, 88.1% of the variance in very fine roots, 81.2% of the variance in fine roots, and 80.7% of the variance in coarse roots. Figure 4 summarizes these R^2^ measures, as well as all significant effects of the regression analyses dependent on land-use types. The vegetation variable ‘richness’, for example, is statistically significant (p-value < 0.15) for the forest land-use type, i.e., the estimate (= 0.960) is shown in the circle denoting forest, but not for agriculturally unused grasslands and intensively used hay meadows. The estimate of ‘Dwarf shrub species’ (= -1.231) is statistically significant (p-value < 0.05) for forests. For each rooting parameter, all significant variables in the left panel are used in the right panel to investigate the rooting parameter for the remaining land-use types. In this respect, the right panel provides explorative findings.

In the *community-level traits,* the variables ‘dominance of small-leaved species’ and ‘very dense rooting species were statistically significant across the root mass measures (Supplementary Appendix S6), for example total root mass estimates are shown in Fig. 4. Overall, 50% of all components/variables showed associations that were statistically significantly different across land use types. This finding underlines the importance of vegetation composition for root mass. The effects of the variables in the *richness* group (richness and dwarf shrub species) were statistically significant in five out of eight possibilities (62.5%) (Supplementary Appendix S6). This kind of importance measure is plotted in Fig. 5 for each variable group.

The estimates of each single component/variable (Supplementary Appendix S6) provide direct effects on root mass. A higher plant cover with even cover distribution between species (*community composition*), a higher ‘number of species’ (*richness*), and a more even distribution among the species (*community-level traits*) went along with an increase in very fine and fine root mass. An increase in the abundance of trees (‘dominance of trees’) correlated with an increase in total root mass but a decrease in fine root mass. The mass of coarse roots was highly correlated with ‘high total plant cover’ and the ‘cover of Juncaceae’; in general, (exception: agriculturally unused grassland), unfavorable growth conditions, higher proportions of silt and soils with a low soil clay content, and reduced root mass. Higher plant water availability of the soil increased very fine root mass (0–1 mm) but reduced coarse root mass.

### Effects on root length

Overall, root length also varied significantly between land-use types (Fig. 3). Land-use types were more important than site conditions (Fig. 5). Extensive management of hay meadows (no nutrient input) or pasturing resulted in higher values of root length on average, while intensive use of meadows or arable lands correlated with low values of root length. The maximum root lengths were measured in lightly used hay meadows, and the smallest values were measured in arable land. In contrast to root mass, thicker roots scarcely contributed to the overall root length (Fig. 3). An exception was observed for arable land, where roots with a diameter of 1–5 mm accounted for 29% of all root length categories.

The explained variance of the regression models for rooting length and length of individual root classes varied: 82.8% for total root length, 82.9% for very fine roots, 86.2% for fine roots and 72.1% for coarse roots (Figs. 4 and 5, Appendices S7 and S10). The vegetation variables, especially the *community composition* and *community-level traits*, had great importance (Fig. 5). A higher abundance of small-leaved species led to a higher rooting length. An increased abundance of very dense rooting species increased root length in agriculturally unused grasslands and forests but decreased root length in intensively used hay meadows. Furthermore, an increased abundance of sedges and lichens increased root length, and Juncaceae abundance decreased root length on average. A higher degree of evenness in the distribution of plant species increased root length, especially for very fine and fine roots.

Unfavorable growth conditions (positive influence), favorable growth conditions (mostly negative influence) and high plant-available water potential of the soil (mostly negative influence) showed statistically significant correlations with the share of finest and coarsest root length and total root length (Appendices S7 and S10).

### Effects on rooting depth

The majority of roots could be found between 0 and 20 cm soil depths in all land-use types (see Fig. 3). Vegetation in intensively used hay meadows and arable lands was less deeply rooted than in extensively used grasslands (pastures and hay meadows) and agriculturally unused grasslands. In forests, the rooting depth might have been deeper than our survey depth of 70 cm. This might have resulted in too low an estimated rooting depth in our sample. More than 87.0% of the variation in rooting depth could be explained by the regression model (Figs. 4 and 5, Appendices S8 and S11). The dominance of sedges and large-leaved species increased rooting depth. The rooting depth with respect to the land-use type forest was significantly influenced by the richness and the cover of some functional groups (e.g., trees, sedge, and lichens). Among the site variables, high elevation growing conditions (negative influence), soil depth (negative influence) and the presence of silty soils (positive influence) were significant.

### Effects of key species on rooting

A PCA demonstrated that key species could be clearly grouped due to similar habitat requirements (see Supplementary Appendix S12), seven components each of which with an eigenvalue larger than 1 were obtained. For instance, the abundances of *Fagus sylvatica*, *Luzula nivea*, *Oxalis acetosella* and *Melittis melissophyllum* were highly correlated with each other (r > 0.77, p-value < 0.001); they were all present in beech forests in northern Italy. *Mentha arvensis*, *Zea mays*, *Rorippa sylvestris*, *Convolvulus arvensis* and *Setaria glauca* were typical species in arable lands. With a regression, the multiple correlations between each component and all vegetation and site components/variables were computed. The high correlation coefficients demonstrated that the information that species may contain is already covered by the vegetation and site variables used depending on the land-use types (Supplementary Appendix 12, column ‘Coefficient of multiple correlations’). This result supported the conclusion that rooting was determined by the ecosystem as a whole and not by individual key species.

## Discussion

We tested our hypotheses with a study to assess different vegetation and site variables on rooting parameters across different types of ecosystems. With our comprehensively unique data set, land-use type-specific results regarding the relationships among biodiversity, site factors and rooting parameters were derived. Thereby, we interpreted biodiversity very broadly and used a variety of variables that capture biodiversity per se as well as community composition, functional diversity and diversity of traits. The sample size was limited due to the huge number of possible explanatory variables.

### Connections of vegetation, land use and site variables on root mass, density and depth

Our results indicate a clear relationship between vegetation composition and root parameters. The number of vegetation variables contributing to the explanation of rooting parameters was large in our study. This is in line with studies in the literature^65,67^. They found that ecosystem processes and functions are driven by the functional diversity of biological communities but are mainly affected by abiotic drivers and direct land-use effects. Following this argumentation, the combination of site and vegetation variables depending on land-use types led to high explanatory power in our analyses. In particular, growth conditions, carbonate bedrock, plant-available water potential, soil depth, intensity of meadow use (which is strongly correlated with fertilization), and for some rooting parameters, the extensive management of meadows, pasture use and forest use described significant associations. Previous investigations indicated a relationship between rooting parameters and site variables (e.g.,^17,25^). Nutrient supply is particularly important; limited nitrogen supply leads to increased root growth for both conifers and angiosperms^13,25^. As a result, plants in nutrient-rich sites possess significantly fewer fine roots, which corresponds to lower root mass and shorter root length in intensively used hay meadows, a result we also found. A similar mechanism also applies to the variable bedrock. Due to rapid eluviation processes and acidic pH values, siliceous soils contain, on average, fewer nutrients^68^. Again, this leads to an increase in root penetration. Apart from these soil-specific factors, changes in growth conditions (e.g., temperature, precipitation, and radiation) influence rooting morphology. According to other authors^69^, elevation, unfavorable growth conditions and decreased soil depth have a negative effect on rooting depth. However, the restriction of our root analysis to a depth of 70 cm likely underestimates the differences in maximal root depth, which was found to vary strongly with land use and other site characteristics (especially in forests^53^).

Community-level maximum rooting depth decreases strongly with increasing elevation, a phenomenon also observed specifically in herbaceous species^57^. According to Nesterova^70^, the maximum rooting depth of *Carlina acaulis*, for instance, decreases by 84% from 450 m a.s.l. (408 cm) to 2070 m a.s.l. (66 cm). Finally, intraspecific or interspecific competition can also lead to changes in root density and distribution. Consequences of partitioning of resources in time and space between individuals of single species or between different species (complementarity of resource use) are, for instance, (a) effects on root growth^37,67^, (b) spatial displacement effects by more competitive species^71^ or (c) changes in root system development^71^. An example of higher partitioning of resources is found in lightly managed hay meadows. This kind of hay meadow is mown once every 1–3 years and is not fertilized, which leads to high species numbers (47–51)^33^. As a consequence, we found significantly more functional types (H’ = 0.60 vs. 0.4 in all other management types; p-value = 0.001) and functional traits (H’ = 1.0 vs. 0.9 in all other management types; p-value = 0.049) in lightly managed hay meadows. However, others^22^ found no evidence that functional diversity in vertical rooting patterns was important for the complementarity effect, which suggests that factors other than belowground resource partitioning may drive the biodiversity–productivity relationship.

### Rooting depends on richness, diversity and functional traits

Variables from the *cover of functional types*, *community-level trai*t and *community composition* groups were significant in determining rooting parameters (Fig. 3, Appendices S6–S11). Variables from the *richness* group significantly improved the explanatory power for root length and rooting depth. This result supports the conclusion that functional diversity rather than species numbers per se strongly determines ecosystem functions^22,25^.

The dominance of small-leaved species with their frequently extensive root systems has significant positive effects on the root mass of the ecosystem. On the other hand, the root mass decreases with increasing number and cover of dwarf shrubs. This highlights that these species adaptations in an ecosystem lead to changes in photosynthetic potential, productivity, and rooting parameters^22^. High total plant cover and high grass and forb cover have positive effects on root length and rooting depth due to their complex belowground structures. The ‘dominance of species with dense rooting systems’ is in most cases positively correlated with root mass and rooting length. Furthermore, vegetation types dominated by large-leaved species had a significantly higher root mass, longer root length and a deeper rooting depth due to their higher proportion of coarse roots. In addition, rooting depth is positively influenced by the number/cover of deeper rooting taxa and groups such as dwarf shrubs, Juncaceae, sedges and large-leaved species. Thus, particular species groups and traits emerge. This result concurs with the mass ratio hypothesis^72^ that ecosystem functions, at a given point in time, are mainly determined by trait values of the dominant contributors to plant biomass or vegetation cover. Surprisingly, an increased abundance of lichens increased root length. Lichens have no roots; therefore, they cannot directly increase root length. However, lichen cover is often associated with shallow poor alpine soils, where root density is high^73^; therefore, the lichen cover probably acts as a placeholder for these soils.

### The role of key species in highly diverse ecosystems

In line with the literature, our results confirm that rooting is determined by the ecosystem as a whole and not by individual key species. Plants in nutrient-rich and wet sites develop significantly fewer fine roots but coarser roots^13,25^. Humid and nutrient-rich sites consistently show more fine and coarse roots. Moreover, forest vegetation not only generates more roots but also penetrates the soil with deeper roots^40,69,74^.

There are other factors besides those considered in our study that may influence the rooting penetration of a stock. In particular, the year-to-year variation in climatic parameters may be an important driving factor in community dynamics^75^. Substantial drought may result in a multiyear decrease in the total belowground biomass. Fiala^76^, for example, described a fourfold decrease in the belowground biomass in the Polygala-Nardetum community over three years as a consequence of considerable drought during the previous vegetation season. This decline was caused by increased root mortality and subsequent decomposition. As we only have repeated measurements of rooting in a few cases and with an insufficient temporal resolution, we could not account for these effects.

Overall, we can demonstrate that high structural and functional diversity of the plant community leads to denser and deeper rooting penetration of soil. This, in turn, affects the entire ecosystem (e.g., by increasing carbon uptake or soil stability), which in turn affects the enhancement of some ecosystem services.

## Conclusions

Rooting parameters are influenced by many complex relationships. Although we had a considerable amount of standardized root data (n = 171) available, these complex relationships could only be partially revealed and not be analyzed in full depth. However, the following valuable conclusions can be drawn:

- Our results show the wide range of rooting patterns in 47 differently used vegetation communities.
- The vegetation composition and its diversity play a central role in the rooting of a soil.
- Rooting is mainly determined by community-level characteristics, composition, and richness.
- Different land-use types determine vegetation composition and thus significantly influence rooting parameters.

To be able to explain these dependencies even better, however, further investigations must follow. It is particularly important that these investigations will be performed in other biomes to be able to map the variability of the site factors. Despite these limitations, this work shows clear correlations among plant composition, diversity and soil rooting. Since vegetation composition is highly dependent on land use and its intensity, it is possible to manage rooting to support services such as densely rooted soil against erosion risks.

## Supplementary Information

Supplementary Information.

## Data Availability

The rooting data base (including all biodiversity variables) was uploaded as online supporting information.
