# Supplementary material for: Evidence for the importance of land use, site characteristics and vegetation composition for rooting in European Alps
Source: Sci Rep. 2021 May 27;11:11246. doi: 10.1038/s41598-021-90652-2 (PMC8159984; doi:10.1038/s41598-021-90652-2)
Supplement: Supplementary file 1 — Supplementary Information. [file 41598_2021_90652_MOESM1_ESM.docx]

**Evidence for the importance of land use, site characteristics and vegetation composition for rooting in Alpine ecosystems**

Erich Tasser, Sonja Gamper, Janette Walde, Nikolaus Obojes, Ulrike Tappeiner

**Appendices**

**Appendix S1**

Sites, number of rooting samples and site characteristics as well as the land-use types within the single sites.
Land-use types: AL = arable land, IM = intensively used hay meadow, LM = lightly managed hay meadow, P = pasture, UG = agriculturally unused grasslands, F = forest; Geology: L = limestone, S = silicate.

| **Project site** | **Rooting samples** (n) | **Altitude** (m a.s.l.) | **Mean temperature** (°C) | **Mean precipitation** (mm) | **Geology** | **Land-use types (n)** |
| --- | --- | --- | --- | --- | --- | --- |
| Monte Bondone (B) | 15 | 1700 | 5.5 | 1189 | L (15) | UG (3), IM (3), P (5), F (4) |
| Igls/Patsch (I) | 15 | 850 | 7.0 | 903 | S (15) | AL (15) |
| Jenesien (J) | 2 | 1489 | 6.3 | 1070 | L (2) | IM (1), P (1) |
| Leutasch (L) | 3 | 1100 | 5.9 | 976 | S (3) | IM (3) |
| Mühlbach (M) | 1 | 1817 | 3.6 | 1445 | S (1) | F (1) |
| Matsch (M2) | 3 | 1810 | 3.7 | 1427 | S (3) | IM (1), LM (1), P (1) |
| Ötz Valley (Ö) | 3 | 1260 | 6.0 | 856 | S (3) | IM (3) |
| Passeier Valley (P) | 21 | 1700 | 4.7 | 1287 | S (21) | IM (6), LM (3), UG (6), F (6) |
|  | 8 | 1287 | 6.8 | 1140 | S (8) | IM (6), P (2) |
|  | 5 | 802 | 9.1 | 854 | S (5) | IM (5) |
| Ritten (R) | 5 | 1627 | 6.1 | 1044 | S (5) | IM (1), LM (1), P (1), F (2) |
| Stubai Valley (S) | 5 | 2700 | 2.5 | 1281 | S (5) | UG (5) |
|  | 27 | 2287 | 3.3 | 1387 | L (8), S (19) | UG (27) |
|  | 31 | 1797 | 4.6 | 1057 | L (6), S (25) | IM (6), LM (7), P (6), F (12) |
|  | 20 | 1000 | 6.4 | 859 | S (20) | IM (20) |
| Toblach (T) | 3 | 1714 | 3.4 | 1153 | S (3) | IM (1), F (2) |
| St. Vigil (V) | 1 | 1889 | 2.2 | 1266 | L (1) | P (1) |
| Ziller Valley (Z) | 3 | 650 | 9.0 | 921 | S (3) | IM (3) |

**Appendix S2**

The analysed plant communities per land-use type, number of samples (n), and mean ($\bar{x}$) and standard deviation (sd) of total root mass (RMtot), total rooting length (RLtot), specific root length (SRL) and main rooting depth (RD95%; depth containing 95% of the total root mass).

| **Plant community** | **n** | **RM_tot_** (g m^-2^) | **RL_tot_** (km m^-2^) | **SRL** (m g^-1^) | **RD_95%_** (cm) |  |
| --- | --- | --- | --- | --- | --- | --- |
|  |  | $\bar{x}\pm sd$^1)^ | $\bar{x}\pm sd$ ^1)^ | $\bar{x}\pm sd$ ^1)^ | $\bar{x}\pm sd$ ^1)^ |  |
| **Arable land** | | | | | |  |
| Rudereto-Secalinetea | 15 | 330 ± 206 | 15.9 ± 10.1 | 50.0 ± 17.1 | 18.4 ± 8.9 |  |
| **Intensively used hay meadow** | | | | | |  |
| Angelico-Cirsietum oleracei | 5 | 445 ± 86 | 31.5 ± 16.7 | 67.0 ± 25.8 | 22.2 ± 11.7 |  |
| Astrantio-Trisetetum | 3 | 612 ± 233 | 26.5 ± 4.1 | 45.9 ± 10.2 | 10.8 ± 2.3 |  |
| Chenopodietum rubri | 2 | 437 / 30 | 33.1 / 2.1 | 75.7 / 71.2 | 10.2 12.9/ |  |
| Convolvulo-Brometum inermis *with Larix* | 1 | 355 | 14.7 | 41.5 | 56.0 |  |
| Festuco-Agrostietum | 9 | 834 ± 340 | 55.9 ± 15.4 | 73.0 ± 19.8 | 10.8 ± 2.0 |  |
| Festuco-Agrostietum with *Larix* | 1 | 1386 | 19.5 | 14.1 | 20.0 |  |
| Filipendulo vulgaris- Arrhenatheretum | 2 | 600 / 520 | 47.3 / 46.9 | 78.8 / 90.2 | 11.1 / 11.9 |  |
| Geranio sylvatici-Trisetetum | 7 | 1038 ± 550 | 58.3 ± 23.0 | 59.0 ± 11.7 | 14.0 ± 2.2 |  |
| Lolietum multiflorae | 3 | 174.0 ± 37 | 11.7 ± 1.5 | 68.4 ± 7.8 | 11.4 ± 2.2 |  |
| Lolietum perennis | 1 | 607 | 31.0 | 50.9 | 7.2 |  |
| Pastinaco-Arrhenateretum | 3 | 525.6± 341.6 | 30.7 ± 10.8 | 74.4 ± 25.0 | 20.2 ± 9.7 |  |
| Poa annua-(Matricaria-Polygonion)-community | 1 | 692 | 35.2 | 50.9 | 8.2 |  |
| Poo-Trisetetum | 4 | 866 ± 411 | 52.3 ± 6.4 | 68.4 ± 23.1 | 14.3 ± 1.4 |  |
| Poo-Trisetetum with *Larix* | 2 | 834 / 3532 | 27.7 / 59.6 | 16.8 / 33.2 | 29.0 / 47.0 |  |
| Prunello-Ranunculetum repentis | 3 | 354 ± 73 | 22.0 ± 7.7 | 63.4 ± 23.4 | 8.4 ± 1.4 |  |
| Ranunculo bulbosi-Arrhenatheretum | 4 | 629 ± 215 | 43.1 ± 8.3 | 72.7 ± 15.9 | 13.2 ± 4.7 |  |
| Ranunculo repentis-Alopecuretum | 2 | 622.9 / 555.4 | 30.5 / 50.9 | 49.0 / 91.7 | 17.3 / 22.0 |  |
| Trisetetum flavescentis | 3 | 798 ± 226 | 54.8 ± 7.4 | 72.2 ± 20.5 | 17.1 ± 6.4 |  |
| **Lightly used hay meadow** | | | | | |  |
| Caricetum davallianae | 1 | 704 | 39.1 | 55.5 | 17.3 |  |
| Festuco-Agrostietum | 1 | 1202.9 | 55.3 | 74.4 | 20.2 |  |
| Pastinaco-Arrhenateretum | 3 | 1773 ± 602 | 83.7 ± 24.0 | 48.2 ± 3.3 | 14.8 ± 2.1 |  |
| Sieversio montanae-Nardetum strictae trifolietosum pratensis | 6 | 1963 ± 517 | 73.9 ± 11.5 | 51.2 ± 10.2 | 30.6 ± 8.8 |  |
| Sieversio montanae-Nardetum strictae with *Larix* | 4 | 1342 ± 346 | 63.6 ± 24.0 | 46.7 ± 8.1 | 23.5 ± 1.7 |  |
| **Pasture** | | | | | |  |
| Crepido-Cynosuretum | 5 | 2181 ± 620 | 69.7 ± 6.5 | 33.7 ± 7.9 | 12.5 ± 2.1 |  |
| Festuco-Cynosuretum | 2 | 656 / 1750 | 55.6 / 82.7 | 84.8 / 47.3 | 27.6 / 9.6 |  |
| Potentillo erectae-Brachypodium pinnate with *Larix* | 1 | 781 | 37.4 | 29.5 | 22.0 |  |
| Seslerio-Caricetum sempervirentis | 6 | 1289 ± 469 | 55.2 ± 19.1 | 42.9 ± 4.1 | 19-4 ± 6.5 |  |
| Sieversio montanae-Nardetum strictae with *Larix* | 2 | 1361 / 1308 | 42.4 / 27.5 | 31.2 / 21.0 | 47.0 / 27.0 |  |
| **Agriculturally unused grasslands** | | | | | | |
| Androsacion alpinae | 2 | 272 / 525 | 21.7 / 39.1 | 77.9 / 74.5 | n.a. |  |
| Caricetum firmae | 3 | 870 ± 816 | 39.7 ± 45.4 | 42.2 ± 12.5 | n.a. |  |
| Caricetum sempervirentis | 8 | 1261 ± 506 | 51.7 ± 16.9 | 42.4 ± 4.6 | 26.0 ± 12.9 |  |
| Dryadetum octopetalae | 3 | 161 ± 48 | 7.4 ± 3.3 | 44.8 ± 12.3 | n.a. |  |
| Junipero-Arctostaphyletum | 3 | 2819 ± 738 | 33.1 ± 5.1 | 12.4 ± 4.2 | 36.6 ± 2.4 |  |
| Minuartia austriaca-(Thlaspion)-community | 2 | 192.7 / 284.0 | 6.9 / 18.3 | 35.8 / 64.4 | n.a. |  |
| Rhododendretum ferruginei | 3 | 781 ± 178 | 16.5 ± 3.1 | 21.6 ± 4.4 | 15.8 ± 3.7 |  |
| Salicetum herbaceae | 1 | 678 | 59.6 | 88.0 | n.a. |  |
| Sieversio montanae-Nardetum strictae typicum | 3 | 1338 ± 432 | 47.5 ± 7.2 | 36.9 ± 6.1 | 14.8 ± 1.0 |  |
| Sieversio montanae-Nardetum strictae vaccinietosum | 6 | 2248 ± 1270 | 37.4 ± 22.2 | 16.5 ± 3.1 | 21.2 ± 7.1 |  |
| Sieversio- Oxyrietum Digynae | 7 | 209 ± 126 | 17.7 ± 12.4 | 81.7 ± 10.9 | n.a. |  |
| **Forests** | | | | | | |
| Calamagrostio villosae-Piceetum | 1 | 2953 | 125.8 | 42.59 | 53.0 |  |
| Dentario pentaphylli-Fagetum | 4 | 3003 ± 1473 | 26.5 ± 6.1 | 9.7 ± 3.2 | 77.1 ± 50.4 |  |
| Homogyno-Piceetum | 3 | 3031 ± 717 | 56.8 ± 21.9 | 18.5 ± 3.9 | 19,5 ± 7.8 |  |
| Larici-Piceetum | 13 | 2328 ± 997 | 35.9 ± 25.1 | 15.8 ± 9.1 | 33.8 ± 12.9 |  |
| Larici-Piceetum, initial stage | 3 | 1979 ± 202 | 74.7 ± 2.2 | 38.0 ± 2.9 | 41.5 ± 4.4 |  |
| Potentillo erectae-Brachypodium pinnate with *Larix* | 1 | 1356 | 40.0 | 47.9 | 57.0 |  |
| Sieversio montanae-Nardetum strictae with *Larix* | 3 | 1332 ± 160 | 55.8 ± 3.5 | 42.2 ± 2,6 | 23.2 ± 2.9 |  |

^1)^ If n = 1 or n = 2 the original values are given; n.a. = not available.

**Appendix S3**

The results of a PCA computed to summarize vegetation variables that describe the canopy at 171 sites into components. 12 components eigenvalue > 1 were extracted which explained 84.0% of total variance (fraction of variance explained by component is given in column ‘% of variance’). Furthermore, two variables with loadings lower than 0.5 for any component were directly employed in the regression models. For each variable and component (Type: C = component; V = variable), the scale type, data range and the factor loading is given. All components were assigned to one of the vegetation composition groups (R=richness, CC=community composition, FT=cover of functional types, CT=community-level traits). The component names were chosen to represent the summarized variables.

| **Component**/variable | **Type** | **Composition group** | **Scale** | **Mean** | **Range** | **PCA % of variance explaind** | **PCA Varimax Rotated Principal Component ^1)^** | **Included** |
| --- | --- | --- | --- | --- | --- | --- | --- | --- |
| ***Richness*** | C | R | metrical | 0 | -1.69 – 2.61 | 10.6 |  | √ |
| Total forb species (n) | V |  | metrical | 17.2 | 1 - 42 |  | 0.952 |  |
| Forb species (n), without legumes | V |  | metrical | 15.5 | 0 - 38 |  | 0.948 |  |
| Vascular plant species (n) | V |  | metrical | 26.1 | 3 - 72 |  | 0.936 |  |
| Total grass species (n) | V |  | metrical | 5.3 | 0 - 13 |  | 0.761 |  |
| *Poaceae* species (n) | V |  | metrical | 4.2 | 0 - 11 |  | 0.670 |  |
| Legume species (n) | V |  | metrical | 1.7 | 0 - 6 |  | 0.624 |  |
| ***Dwarf shrub species (n)*** | *V* | R | metrical | 0 | 0 - 8 |  |  | √ |
| ***Community composition*** | C | CC | metrical | 0 | -3.04 - 1.15 | 19.5 |  | √ |
| Total Evenness- index (species, functional types and functional traits) | V |  | metrical | 0.84 | 0.49 – 0.96 |  | 0.945 |  |
| Evenness- index of species | V |  | metrical | 0.74 | 0.09 - 0.99 |  | 0.933 |  |
| Evenness- index of functional traits | V |  | metrical | 0.83 | 0.40 - 0.96 |  | 0.927 |  |
| Total Shannon-Wiener- index (species, functional types and functional traits) | V |  | metrical | 1.04 | 0.60 – 1.22 |  | 0.919 |  |
| Shannon-Wiener- index of functional types | V |  | metrical | 0.45 | 0.03 - 0.78 |  | 0.919 |  |
| Evenness- index of functional types | V |  | metrical | 0.68 | 0.08 - 0.99 |  | 0.859 |  |
| Shannon-Wiener- index of species | V |  | metrical | 1.02 | 0.09 - 1.69 |  | 0.812 |  |
| Dominance of single species (cover > 87%) (y/n) | V |  | dichotomic | 0.1 | 0 - 1 |  | -0.805 |  |
| Plant cover variance | V |  | metrical | 122.4 | 0.3 - 1406.2 |  | -0.748 |  |
| Shannon-Wiener- index of functional traits | V |  | metrical | 0.92 | 0.44 - 1.10 |  | 0.669 |  |
| Dominance of single species (cover > 62%) (y/n) | V |  | dichotomic | 0.3 | 0 - 1 |  | -0.665 |  |
| Cover of very large species (plant height >90cm) (%) | V |  | metrical | 14.3 | 0 - 97.4 |  | -0.647 |  |
| Cover of medium-size species (plant height 20-40cm) (%) | V |  | metrical | 25.3 | 0 - 73.2 |  | 0.516 |  |
| ***Dominance of cryptogams*** | C | FT | metrical | 0 | -1.06 – 4.97 | 7.9 |  | √ |
| Moss cover (%) | V |  | metrical | 3.1 | 0 - 63.0 |  | 0.934 |  |
| Cryptogam cover (%) | V |  | metrical | 3.7 | 0 - 63.0 |  | 0.912 |  |
| Moss species (n) | V |  | metrical | 0.5 | 0 - 6 |  | 0.794 |  |
| Cryptogam species (n) | V |  | metrical | 0.7 | 0 - 6 |  | 0.680 |  |
| ***Dominance of trees*** | C | FT | metrical | 0 | -2.80 – 3.37 | 7.0 |  | √ |
| Cover of deep-rooting species (main rooting depth 30-100cm) (%) | V |  | metrical | 28.4 | 0 - 104.4 |  | 0.778 |  |
| Tree species (n) | V |  | metrical | 0.6 | 0 - 6 |  | 0.750 |  |
| Tree cover (%) | V |  | metrical | 4.7 | 0 - 78.8 |  | 0.711 |  |
| Cover of mean deep-rooting species (main rooting depth 10-30cm) (%) | V |  | metrical | 60.6 | 0 - 215.6 |  | -0.624 |  |
| Fern species (n) | V |  | metrical | 0.3 | 0 - 6 |  | 0.551 |  |
| ***Dominance of lichens*** | C | FT | metrical | 0 | -1.42 – 4.61 | 6.0 |  | √ |
| Lichen species (n) | V |  | metrical | 0.2 | 0 - 3 |  | 0.928 |  |
| Lichen cover (%) | V |  | metrical | 0.5 | 0 - 12.8 |  | 0.925 |  |
| Cover of medium-size species (plant height 20-40cm) (%) | V |  | metrical | 25.3 | 0 - 73.2 |  | 0.581 |  |
| Dwarf shrub cover (%) | V |  | metrical | 13.4 | 0 - 107.3 |  | 0.525 |  |
| Cryptogam species (n) | V |  | metrical | 0.7 | 0 - 6 |  | 0.504 |  |
| ***Dominance of forbs*** | C | FT | metrical | 0 | -1.93 – 4.46 | 5.5 |  | √ |
| Total forb cover (%) | V |  | metrical | 56.0 | 1.8 - 197.3 |  | 0.747 |  |
| Legume cover (%) | V |  | metrical | 10.0 | 0 - 78.8 |  | 0.712 |  |
| Forb cover (%), without legumes | V |  | metrical | 46.0 | 1.8 - 126.7 |  | 0.647 |  |
| Mean species cover (%) | V |  | metrical | 6.1 | 1.4 - 36.3 |  | 0.620 |  |
| ***Dominance of sedges*** | C | FT | metrical | 0 | -1.44 – 5.10 | 3.6 |  | √ |
| Cover of *Cyperaceae* (%) | V |  | metrical | 3.3 | 0 - 50.8 |  | 0.872 |  |
| *Cyperaceae* species (n) | V |  | metrical | 0.6 | 0 - 3 |  | 0.820 |  |
| ***High total cover with a high grass cover*** | C | FT | metrical | 0 | -1.22 – 5.25 | 6.0 |  | √ |
| Cover of *Poaceae* (%) | V |  | metrical | 36.7 | 0 - 174.0 |  | 0.674 |  |
| Total grass cover (%) | V |  | metrical | 41.6 | 0 - 174.0 |  | 0.667 |  |
| Total vegetation coverage (%) | V |  | metrical | 86.0 | 9 - 100 |  | 0.651 |  |
| Cover of large species (plant height 40-90cm) (%) | V |  | metrical | 40.3 | 0 - 99.0 |  | 0.628 |  |
| Cover of small species (plant height < 20cm) (%) | V |  | metrical | 34.4 | 0.3 - 100.0 |  | -0.616 |  |
| ***Cover of Juncaceae (%)*** | *V* | FT | metrical | 1.6 | 0 - 38.3 |  |  | **√** |
| ***Dominance of large-leaved species*** | C | CT | metrical | 0 | -2.16 – 4.48 | 3.4 |  | √ |
| Cover of large-leaved species (leaf area > 70cm²) (%) | V |  | metrical | 8.6 | 0 - 97.4 |  | 0.829 |  |
| Fern cover (%) | V |  | metrical | 0.7 | 0 - 20.5 |  | 0.534 |  |
| ***Dominance of small-leaved species*** | C | CT | metrical | 0 | -3.28 – 1.97 | 5.9 |  | √ |
| Cover of mean-leaved species (leaf area 10-70cm²) (%) | V |  | metrical | 26.7 | 0 - 96.4 |  | -0.802 |  |
| Cover of small-leaved species (leaf area < 10cm²) (%) | V |  | metrical | 64.7 | 2 - 100.0 |  | 0.741 |  |
| Cover of species with sparse rooting density (%) | V |  | metrical | 15.8 | 0 - 83.3 |  | -0.547 |  |
| ***Dominance of very dense rooting species*** | C | CT | metrical | 0 | -2.82 – 4.83 | 2.8 |  | √ |
| Cover of species with very dense rooting density (%) | V |  | metrical | 2.1 | 0 - 54.7 |  | 0.623 |  |
| Cover of very deep-rooting species (main rooting depth >100cm) (%) | V |  | metrical | 2.2 | 0 - 38.0 |  | -0.501 |  |
| ***Mean deep rooting species with medium dense rooting density*** | C | CT | metrical | 0 | -4.90 – 2.01 | 5.6 |  | √ |
| Cover of species with medium dense rooting density (%) | V |  | metrical | 60.1 | 0 - 100.0 |  | 0.856 |  |
| Cover of species with dense rooting density (%) | V |  | metrical | 22.0 | 0 - 100.0 |  | -0.795 |  |
| Cover of shallow rooting species (main rooting depth < 10cm) (%) | V |  | metrical | 13.8 | 0 - 80.6 |  | -0.718 |  |
| Cover of mean deep-rooting species (main rooting depth 10-30cm) (%) | V |  | metrical | 60.6 | 0 - 215.6 |  | 0.503 |  |

**^1^**) Extraction Method: Principal Component Analysis. Rotation Method: Varimax with Kaiser Normalization. Only factor loadings > 0.5 or < -0.5 are shown.

**Appendix S4**

The results of a PCA computed to summarise site variables into components. Five components with an eigenvalue > 1 were extracted which explained 77.6% of total variance (fraction of variance explained by a component is given in column ‘% of variance’). Furthermore, one variable with loadings of less than 0.5 in absolute value was directly employed in the regression model. C = component; V = variable.

| **Component / variable** | **Type** | **Scale** | **Mean** | **Range** | **PCA % of variance explaind** | **PCA Varimax Rotated Principal Component ^1)^** | **Included** | |
| --- | --- | --- | --- | --- | --- | --- | --- | --- |
| **High elevation growing conditions** | C | metrical | 0 | -2.07 – 2.17 | 28.2 |  | √ | |
| Bulk density (g·cm^−3^) | V | metrical | 2.22 | 0.87 - 6.64 |  | -0.871 |  |  |
| Mean annual temperature (°C) | V | metrical | 5.2 | 2.2 - 9.4 |  | -0.808 |  |  |
| Soil organic C (%) | V | metrical | 10.4 | 2.2 - 27.0 |  | 0.786 |  |  |
| Soil organic matter (%) | V | metrical | 17.2 | 3.2 - 45.7 |  | 0.786 |  |  |
| Mean total precipitation (mm) | V | metrical | 1121 | 806 - 1644 |  | 0.775 |  |  |
| Total pore volume (%) | V | metrical | 64.0 | 25.3 - 89.0 |  | 0.725 |  |  |
| Total particle density (g·cm^−3^) | V | metrical | 0.80 | 0.21 - 1.45 |  | -0.514 |  |  |
| **Favorable growth conditions** | C | metrical | 0 | -2.11 – 1.92 | 17.8 |  | √ | |
| Soil productivity or fertility (Ellenberg's indicator value N) | V | metrical | 4.1 | 1.3 - 6.9 |  | 0.914 |  |  |
| Soil acidity (Ellenberg's indicator value R) | V | metrical | 5.4 | 1.5 - 8.1 |  | 0.802 |  |  |
| Elevation (m a.s.l.) | V | metrical | 1582.9 | 650 - 2700 |  | -0.790 |  |  |
| Temperature (Ellenberg's indicator value T) | V | metrical | 3.9 | 1.2 - 6.3 |  | 0.713 |  |  |
| **Silty soil** | C | metrical | 0 | -2.35 – 1.88 | 13.0 |  | √ | |
| Sand (%) | V | metrical | 36.8 | 0.8 - 79.7 |  | -0.943 |  |  |
| Silt (%) | V | metrical | 48.9 | 3.9 - 88.1 |  | 0.926 |  |  |
| **Carbonat bedrock / low soil clay content** | C | metrical | 0 | -3.16 – 2.34 | 10.5 |  | √ | |
| Carbonat bedrock | V | dichotomic | 0.2 | 0 - 1 |  | 0.822 |  |  |
| Clay (%) | V | metrical | 13.2 | 0.0 - 48.2 |  | -0.595 |  |  |
| **High plant available water content** | C | metrical | 0 | -3.48 – 3.52 | 8.1 |  | √ | |
| Soil humidity (Ellenberg's indicator value F) | V | metrical | 5.1 | 3.8 - 8.8 |  | 0.665 |  |  |
| Clay (%) | V | metrical | 13.2 | 0.0 - 48.2 |  | -0.545 |  |  |
| Total particle density (g·cm^−3^) | V | metrical | 0.80 | 0.21 - 1.45 |  | 0.520 |  |  |
| **Soil depth (mm)** | V | metrical | 194.8 | 12 - 770 |  |  | √ |  |

**^1^**) Extraction Method: Principal Component Analysis. Rotation Method: Varimax with Kaiser Normalization. Only factor loadings > 0.5 or < -0.5 are shown.

**Appendix S5**

Results of the F-tests indicating (a) the necessity for different level and slope effects according to land use in the regression models (Eq. (3)), i.e. $H_{0}:LU=0 and \beta_{LU}=0 and \gamma_{LU}=0$, and (b) indicating the necessity for different slope effects according to land use in the regression models (Eq. (3)), i.e. $H_{0}: \beta_{LU}=0 and \gamma_{LU}=0$. Of all models, 50% show statistically significant relationships different with respect to land use indicating an important explanatory contribution.

|  | **(a) different level and slope effects** | | **(b) only different slope effects** | |
| --- | --- | --- | --- | --- |
| **dependent variable** | **F-test** | **significance** | **F-test** | **significance** |
| total root mass | F( 43, 48) = 2.10 | 0.0066 | F( 41, 48) = 2.04 | 0.0091 |
| root mass of root diameter class 0-1 mm | F( 43, 49) = 2.39 | 0.0017 | F( 41, 49) = 2.04 | 0.0085 |
| root mass of root diameter class 1-5 mm | F( 43, 49) = 1.53 | 0.076 | F( 41, 49) = 1.58 | 0.0617 |
| root mass of root diameter class 5-20 mm | F( 43, 49) = 2.11 | 0.0061 | F( 41, 49) = 2.09 | 0.007 |
| total root length | F( 43, 47) = 1.58 | 0.0628 | F( 41, 47) = 1.56 | 0.0707 |
| root length of root diameter class 0-1mm | F( 43, 47) = 1.59 | 0.0615 | F( 41, 47) = 1.57 | 0.0692 |
| root length of root diameter class 1-5 mm | F( 43, 47) = 2.16 | 0.0052 | F( 41, 47) = 2.20 | 0.0047 |
| root length of root diameter class 5-20 mm | F( 43, 49) = 1.71 | 0.0344 | F( 41, 49) = 1.59 | 0.0599 |
| root depth (50% of roots above) | F( 43, 48) = 2.13 | 0.0058 | F( 41, 48) = 0.89 | 0.6420 |
| root depth (90% of roots above) | F( 43, 48) = 1.66 | 0.0441 | F( 41, 48) = 0.96 | 0.5553 |
| root depth (95% of roots above) | F( 43, 48) = 1.52 | 0.0796 | F( 41, 48) = 1.21 | 0.2648 |

**Appendix S6**

Regression analyses showing the effects of biodiversity, land use and site characteristics on different classes of root mass (RM). Standardized coefficients for all variables with p-value < 0.15 are displayed. Valid sample size for all land-use types (n), the determination coefficient (R²) and the adjusted determination coefficient (adj. R²) are provided. Land-use type: IM=intensively used hay meadow, UG= agriculturally unused grasslands, F=forest use; biodiversity group membership: R=richness, CC=community composition, FT=cover of functional types, CT=community-level traits; S=site variable

| **Vegetation and site variables** | **RM_tot_** | | | **RM_0-1_** | | | **RM_1-5_** | | | **RM_5-20_** | | |
| --- | --- | --- | --- | --- | --- | --- | --- | --- | --- | --- | --- | --- |
|  | **IM** | **UG** | **F** | **IM** | **UG** | **F** | **IM** | **UG** | **F** | **IM** | **UG** | **F** |
| Richness (R) |  |  | 0.960^+^ |  |  |  |  |  |  |  |  | 1.471** |
| Dwarf shrub species (R) |  |  | -1.231* |  |  | -0.742* |  |  | -1.435*** |  |  |  |
| Community composition (CC) |  |  |  |  |  | 0.989** |  | 0.873* |  |  | 0.690^+^ |  |
| High total plant cover with a high grass cover (FT) |  |  |  |  |  |  |  | -1.334^+^ |  |  | 0.980^+^ | 0.759^+^ |
| Dominance of cryptogams (FT) |  |  |  |  |  | 0.848** |  | 0.437 |  |  | 0.437^+^ |  |
| Dominance of trees (FT) |  | 1.266^+^ |  |  |  |  |  |  | -0.841^+^ |  |  |  |
| Dominance of sedges (FT) |  |  |  |  |  | 0.989* |  |  |  |  | 1.026^+^ |  |
| Dominance of lichens (FT) |  |  |  |  |  |  |  |  | 2.023* |  |  |  |
| Dominance of forbs (FT) |  |  |  |  |  |  |  |  |  |  |  |  |
| Cover of *Juncaceae* (FT) |  |  |  |  |  |  |  |  |  |  | 0.677^+^ | 0.604^+^ |
| Dominance of small-leaved species (CT) | 0.279^+^ |  |  | 0.366** |  | 0.756^+^ |  | 2.133^+^ |  |  |  |  |
| Deep rooting species/medium dense rooting density (CT) |  |  |  | 0.282* |  | 0.753^+^ |  |  |  |  | 0.822** |  |
| Dominance of large-leaved species (CT) |  |  |  |  |  |  |  |  | 0.523^+^ |  | 0.315^+^ | 1.006*** |
| Dominance of very dense rooting species (CT) |  |  |  |  | 2.154** | 0.785^+^ |  | 1.573^+^ |  |  | -1.846^**^ |  |
| Unfavorable growth conditions (S) |  |  |  |  |  |  |  | -1.062^+^ |  |  | 0.690^+^ | -2.162** |
| Favorable growth conditions (S) |  |  |  |  | 1.354^+^ |  |  |  |  |  |  |  |
| Silty soil (S) |  |  |  |  |  | -1.402** |  |  |  |  |  |  |
| Carbonat bedrock/low soil clay content (S) |  |  | -0.606^+^ |  |  |  |  |  |  |  | 1.732** | -0.982** |
| High plant available water content (S) |  |  |  |  |  | 1.540*** |  |  |  |  | -0.980** |  |
| Soil depth (S) |  |  |  |  |  |  |  |  |  |  | -0.474** |  |
| Interaction effects with corresponding other land-use types in each regression included. | | | | | | | | | | | | |
| n | 111 | | | 112 | | | 112 | | | 112 | | |
| R² | 0.882 | | | 0.881 | | | 0.812 | | | 0.807 | | |
| adj. R² | 0.730 | | | 0.730 | | | 0.573 | | | 0.562 | | |

Note: *** = p<0.01; ** = p<0.05; * = p<0.10; ^+^ = p<0.15. Significances are based on bootstrapped standard errors.

**Appendix S7**

Regression analyses showing the effects of biodiversity, land use and site characteristics on different classes of root length (RL). Standardized coefficients for all variables with p-value < 0.15 are displayed. Valid sample size for all land-use types (n), the determination coefficient (R²) and the adjusted determination coefficient (adj. R²) are provided. Land-use type: IM=intensively used hay meadow, UG=agriculturally unused grasslands, F=forest use; biodiversity group membership: R=richness, CC=community composition, FT=cover of functional types, CT=community-level traits; S=site variable

| **Vegetation and site variables** | **RL_tot_** | | | **RL_0-1_** | | | **RL_1-5_** | | | **RL_5-20_** | | |
| --- | --- | --- | --- | --- | --- | --- | --- | --- | --- | --- | --- | --- |
|  | **IM** | **UG** | **F** | **IM** | **UG** | **F** | **IM** | **UG** | **F** | **IM** | **UG** | **F** |
| Richness (R) |  |  |  |  |  |  |  |  |  |  |  |  |
| Dwarf shrub species (R) |  |  |  |  |  |  |  |  | -1.505*** |  |  |  |
| Community composition (CC) |  |  |  |  |  |  |  | 0.691^+^ |  |  |  |  |
| High total plant cover with a high grass cover (FT) |  |  |  |  |  |  |  |  |  |  |  | 1.073^+^ |
| Dominance of cryptogams (FT) |  |  |  |  |  |  |  |  |  |  | 0.662^+^ |  |
| Dominance of trees (FT) |  |  |  |  |  |  |  |  | -0.996* |  |  |  |
| Dominance of lichens (FT) | 1.643** |  |  | 1.644** |  |  |  |  | 1.986* |  |  |  |
| Dominance of forbs (FT) |  |  |  |  |  |  |  |  |  |  |  |  |
| Dominance of sedges (FT) |  |  |  |  |  |  | 0.258* |  |  |  | 1.530^+^ |  |
| Cover of *Juncaceae* (FT) | -0.778^+^ |  |  | -0.778^+^ |  |  |  |  |  |  | 1.508** |  |
| Dominance of small-leaved species (CT) | 0.699*** |  |  | 0.697*** |  |  |  | 1.697^+^ |  |  |  | 1.142^+^ |
| Deep rooting species/medium dense rooting density (CT) | 0.594*** |  |  | 0.593*** |  |  |  |  |  |  |  | 1.017^+^ |
| Dominance of large-leaved species (CT) |  |  |  |  |  |  |  |  | 0.579** |  |  | 1.116*** |
| Dominance of very dense rooting species (CT) | -0.585* | 2.195* |  | -0.585* | 2.187* |  |  |  |  |  |  | 1.279^+^ |
| Unfavorable growth conditions (S) | 0.362^+^ |  |  | 0.365^+^ |  |  |  |  |  | 0.478* |  |  |
| Favorable growth conditions (S) | -0.700** |  |  | -0.703** |  |  |  |  |  |  |  | 2.598** |
| Silty soil (S) |  |  |  |  |  |  |  |  |  |  |  |  |
| Carbonat bedrock/low soil clay content (S) |  |  |  |  |  |  |  |  |  |  |  | -1.311** |
| High plant available water content (S) |  |  | 1.431* |  |  | 1.439* | -0.375^+^ |  |  |  |  |  |
| Soil depth (S) |  |  |  |  |  |  |  |  |  |  |  |  |
| Interaction effects with corresponding other land-use types included | | | | | | | | | | | | |
| n | 110 | | | 110 | | | 110 | | | 112 | | |
| R² | 0.828 | | | 0.829 | | | 0.862 | | | 0.721 | | |
| adj. R² | 0.601 | | | 0.603 | | | 0.681 | | | 0.369 | | |

Note: *** = p<0.01; ** = p<0.05; * = p<0.10; ^+^ = p<0.15. Significances are based on bootstrapped standard errors.

**Appendix S8**

Regression analyses showing the effects of biodiversity, land use and site characteristics on different classes of root depth (RD). Standardized coefficients for all variables with p-value < 0.15 are displayed. Valid sample size for all land-use types (n), the determination coefficient (R²) and the adjusted determination coefficient (adj. R²) are provided. Land-use type: IM=intensively used hay meadow, UG=agriculturally unused grasslands, F=forest use; biodiversity group membership: R=richness, CC=community composition, FT=cover of functional types, CT=community-level traits; S=site variable

| **Vegetation and site variables** | **RD_95%_** | | | **RD_90%_** | | | **RD_50%_** | | |
| --- | --- | --- | --- | --- | --- | --- | --- | --- | --- |
|  | **IM** | **UG** | **F** | **IM** | **UG** | **F** | **IM** | **UG** | **F** |
| Richness (R) |  |  | -1.262* |  |  |  |  |  |  |
| Dwarf shrub species (R) |  |  |  |  |  |  |  |  |  |
| Community composition (CC) |  |  |  |  |  |  |  |  |  |
| High total plant cover with a high grass cover (FT) |  |  |  |  |  |  |  |  |  |
| Dominance of cryptogams (FT) |  |  |  |  |  |  |  |  |  |
| Dominance of trees (FT) |  |  | -1.100^+^ |  |  |  |  |  |  |
| Dominance of lichens (FT) |  |  | 2.612** |  |  |  |  |  |  |
| Dominance of forbs (FT) |  |  |  |  |  |  |  |  |  |
| Dominance of sedges (FT) | 0.325** |  | 0.672** | 0.357*** |  |  | 0.341*** |  |  |
| Cover of *Juncaceae* (FT) |  |  | -0.891* |  |  |  |  |  |  |
| Dominance of small-leaved species (CT) |  |  |  |  |  |  |  |  |  |
| Deep rooting species/medium dense rooting density (CT) |  |  |  |  |  |  |  |  |  |
| Dominance of large-leaved species (CT) |  | 0.754*** |  |  | 0.716*** | 0.520* |  | 0.650*** |  |
| Dominance of very dense rooting species (CT) |  |  |  |  |  |  |  |  |  |
| Unfavorable growth conditions (S) |  |  |  |  | -0.984* |  |  | -0.971* |  |
| Favorable growth conditions (S) |  |  |  |  |  |  |  |  |  |
| Silty soil (S) |  |  | 1.615^+^ |  |  |  |  |  |  |
| Carbonat bedrock/low soil clay content (S) |  |  |  |  |  |  |  |  |  |
| High plant available water content (S) |  |  |  |  |  |  |  |  |  |
| Soil depth (S) |  |  | -2.819** |  |  | -2.554** |  |  |  |
| Interaction effects with corresponding other land-use types included | | | | | | | | | |
| n | 111 | | | 111 | | | 111 | | |
| R² | 0.872 | | | 0.880 | | | 0.891 | | |
| adj. R² | 0.706 | | | 0.725 | | | 0.751 | | |

Note: *** = p<0.01; ** = p<0.05; * = p<0.10; ^+^ = p<0.15. Significances are based on bootstrapped standard errors.

**Appendix S9**

Regression analyses showing the estimated effects of biodiversity, land use and site characteristics on different classes of root mass (RM). Standardized coefficients for all variables with p-value < 0.15 are displayed. Valid sample size for all land-use types (n), the determination coefficient (R²) and the adjusted determination coefficient (adj. R²) are provided. Only the variables with p<0.15 from the analyses concerning IM, UG and F are used (cf. Appendix 6). and use type: AL=arable land, P=pasture, LM=lightly managed hay meadow; biodiversity group membership: R=richness, CC=community composition, FT=cover of functional types, CT=community-level traits; S=site variable.

| **Vegetation and site variables** | **RM_tot_** | | | **RM_0-1_** | | | **RM_1-5_** | | | **RM_5-20_** | | |
| --- | --- | --- | --- | --- | --- | --- | --- | --- | --- | --- | --- | --- |
|  | **AL** | **P** | **LM** | **AL** | **P** | **LM** | **AL** | **P** | **LM** | **AL** | **P** | **LM** |
| Richness (R) |  |  |  | --- | --- | --- | --- | --- | --- | 0.834** | 0.606** |  |
| Dwarf shrub species (R) | --- | -0.244^+^ |  | --- | -0.635*** | -0.777*** | --- |  |  | --- | --- | --- |
| Community composition (CC) | --- | --- | --- |  |  |  | 1.093*** | -2.000** | -3.212*** | --- | --- | --- |
| High total plant cover with a high grass cover (FT) | --- | --- | --- | --- | --- | --- | 0.549^+^ |  |  |  |  |  |
| Dominance of cryptogams (FT) | --- | --- | --- | 0.887** | 1.659*** | 1.299*** | --- | --- | --- | --- | --- | --- |
| Dominance of trees (FT) |  |  |  | --- | --- | --- | 0.719* | 0.944*** | 0.881** | --- | --- | --- |
| Dominance of lichens (FT) | --- | --- | --- | --- | --- | --- |  |  | 1.098^+^ | --- | --- | --- |
| Dominance of forbs (FT) | --- | --- | --- | --- | --- | --- | --- | --- | --- | --- | --- | --- |
| Dominance of sedges (FT) | --- | --- | --- |  |  | -0.333** | --- | --- | --- | --- | --- | --- |
| Cover of *Juncaceae* (FT) | --- | --- | --- | --- | --- | --- | --- | --- | --- | 0.907** | 0.761** | 0.892** |
| Dominance of small-leaved species (CT) | 0.251*** |  |  |  | 1.476*** | 1.431*** |  |  | -0.766^+^ | --- | --- | --- |
| Deep rooting species/medium dense rooting density (CT) | --- | --- | --- |  | 1.292*** | 0.614*** | --- | --- | --- |  | 0.752** |  |
| Dominance of large-leaved species (CT) | --- | --- | --- | --- | --- | --- |  | 0.972* |  |  | 1.050** |  |
| Dominance of very dense rooting species (CT) | --- | --- | --- |  |  | 1.061** |  | 1.181*** | 1.320** |  |  |  |
| Unfavorable growth conditions (S) | --- | --- | --- | --- | --- | --- | -0.596** | -0.413* | -0.855** |  | -0.515* |  |
| Favorable growth conditions (S) | --- | --- | --- | -0.559^+^ | 0.642^+^ | 0.682^+^ | --- | --- | --- | --- | --- | --- |
| Silty soil (S) | --- | --- | --- |  |  | -0.618*** | --- | --- | --- | --- | --- | --- |
| Carbonat bedrock/low soil clay content (S) |  |  |  | --- | --- | --- | --- | --- | --- |  |  | 0.649** |
| High plant available water content (S) | --- | --- | --- |  | 0.474* | 0.378^+^ | --- | --- | --- |  |  |  |
| Soil depth (S) | --- | --- | --- | --- | --- | --- | --- | --- | --- |  | -0.319** |  |
| Interaction effects with corresponding other land-use types included | | | | | | | | | | | | |
| n | 151 | | | 152 | | | 152 | | | 152 | | |
| R² | 0.706 | | | 0.815 | | | 0.771 | | | 0.791 | | |
| adj. R² | 0.619 | | | 0.680 | | | 0.628 | | | 0.633 | | |

Note: *** = p<0.01; ** = p<0.05; * = p<0.10; ^+^ = p<0.15; ‘---' = if variable was not used in the regression. Significances are based on bootstrapped standard errors.

**Appendix S10**

Regression analyses showing the estimated effects of biodiversity, land use and site characteristics on different classes of root length (RL). Standardized coefficients for all variables with p-value < 0.15 are displayed. Valid sample size for all land-use types (n), the determination coefficient (R²) and the adjusted determination coefficient (adj. R²) are provided. Only the variables with p<0.15 from the analyses concerning IM, UG and F are used (cf. Appendix 7). Land-use type: AL=arable land, P=pasture, LM=lightly managed hay meadow; biodiversity group membership: R=richness, CC=community composition, FT=cover of functional types, CT=community-level traits; S=site variable.

| **Vegetation and site variables** | **RL_tot_** | | | **RL_0-1_** | | | **RL_1-5_** | | | **RL_5-20_** | | |
| --- | --- | --- | --- | --- | --- | --- | --- | --- | --- | --- | --- | --- |
|  | **AL** | **P** | **LM** | **AL** | **P** | **LM** | **AL** | **P** | **LM** | **AL** | **P** | **LM** |
| Richness (R) | --- | --- | --- | --- | --- | --- | --- | --- | --- | --- | --- | --- |
| Dwarf shrub species (R) | --- | --- | --- | --- | --- | --- | --- |  |  | --- | --- | --- |
| Community composition (CC) | --- | --- | --- | 0.379^+^ |  |  | 0.613*** | -1.173^+^ |  | --- | --- | --- |
| High total plant cover with a high grass cover (FT) | --- | --- | --- | --- | --- | --- | --- | --- | --- |  | 1.649*** |  |
| Dominance of cryptogams (FT) | --- | --- | --- | --- | --- | --- | --- | --- | --- |  |  |  |
| Dominance of trees (FT) | --- | --- | --- | --- | --- | --- |  | 0.462* | 0.455^+^ | --- | --- | --- |
| Dominance of lichens (FT) |  |  | 1.377*** |  |  | 1.346*** |  |  | 1.156** | --- | --- | --- |
| Dominance of forbs (FT) | --- | --- | --- | --- | --- | --- | --- | --- | --- | --- | --- | --- |
| Dominance of sedges (FT) | --- | --- | --- | --- | --- | --- |  |  |  |  |  |  |
| Cover of *Juncaceae* (FT) |  |  |  |  |  |  | --- | --- | --- | 1.823*** | 0.909** | 0.542^+^ |
| Dominance of small-leaved species (CT) |  |  |  |  |  |  |  |  |  |  | -2.056*** |  |
| Deep rooting species/medium dense rooting density (CT) |  | 0.478^+^ | 0.661** |  | 0.497* | 0.609** | --- | --- | --- | --- | --- | --- |
| Dominance of large-leaved species (CT) | --- | --- | --- | --- | --- | --- | 1.183** |  |  | 2.036** | 0.816* |  |
| Dominance of very dense rooting species (CT) |  |  |  | -0.272^+^ |  |  | --- | --- | --- |  | 0.913^+^ | 1.947*** |
| Unfavorable growth conditions (S) |  | -0.352* | -0.629** |  |  | -0.676** | --- | --- | --- |  |  |  |
| Favorable growth conditions (S) | -0.553* |  |  | -0.566+ |  |  | --- | --- | --- |  |  | 1.572** |
| Silty soil (S) | --- | --- | --- | --- | --- | --- | --- | --- | --- | --- | --- | --- |
| Carbonat bedrock/low soil clay content (S) | --- | --- | --- | --- | --- | --- | --- | --- | --- |  | 0.613* |  |
| High plant available water content (S) |  |  |  |  |  |  |  |  |  | --- | --- | --- |
| Soil depth (S) | --- | --- | --- | --- | --- | --- | --- | --- | --- | --- | --- | --- |
| Interaction effects with corresponding other land-use types included | | | | | | | | | | | | |
| n | 150 | | | 150 | | | 147 | | | 149 | | |
| R² | 0.794 | | | 0.838 | | | 0.768 | | | 0.704 | | |
| adj. R² | 0.681 | | | 0.732 | | | 0.639 | | | 0.472 | | |

Note: *** = p<0.01; ** = p<0.05; * = p<0.10; ^+^ = p<0.15; ‘---' = if variable was not used in the regression. Significances are based on bootstrapped standard errors.

**Appendix S11**

Regression analyses showing the estimated effects of biodiversity, land use and site characteristics on different classes of root depth (RD). Standardized coefficients for all variables with p-value < 0.15 are displayed. Valid sample size for all land-use types (n), the determination coefficient (R²) and the adjusted determination coefficient (adj. R²) are provided. Only the variables with p<0.15 from the analyses concerning IM, UG and F are used (cf. Appendix 8). Land-use type: AL=arable land, P=pasture, LM=lightly managed hay meadow; biodiversity group membership: R=richness, CC=community composition, FT=cover of functional types, CT=community-level traits; S=site variable.

| **Vegetation and site variables** | **RD_95%_** | | | **RD_90%_** | | | **RD_50%_** | | |
| --- | --- | --- | --- | --- | --- | --- | --- | --- | --- |
|  | **AL** | **P** | **LM** | **AL** | **P** | **LM** | **AL** | **P** | **LM** |
| Richness (R) |  | 0.448* | 0.479** | --- | --- | --- | --- | --- | --- |
| Dwarf shrub species (R) | --- | --- | --- | --- | --- | --- |  |  |  |
| Community composition (CC) | --- | --- | --- | --- | --- | --- |  |  |  |
| High total plant cover with a high grass cover (FT) | --- | --- | --- | --- | --- | --- | --- | --- | --- |
| Dominance of cryptogams (FT) | --- | --- | --- | --- | --- | --- |  |  |  |
| Dominance of trees (FT) |  |  |  | --- | --- | --- |  |  |  |
| Dominance of lichens (FT) |  | -1.491* |  | --- | --- | --- | --- | --- | --- |
| Dominance of forbs (FT) | --- | --- | --- | --- | --- | --- | --- | --- | --- |
| Dominance of sedges (FT) | 0.700** |  |  |  |  |  |  | 0.294^+^ |  |
| Cover of *Juncaceae* (FT) |  |  |  | --- | --- | --- |  |  |  |
| Dominance of small-leaved species (CT) | --- | --- | --- | --- | --- | --- | --- | --- | --- |
| Deep rooting species/medium dense rooting density (CT) | --- | --- | --- | --- | --- | --- |  |  |  |
| Dominance of large-leaved species (CT) |  |  |  |  |  |  |  |  |  |
| Dominance of very dense rooting species (CT) | --- | --- | --- | --- | --- | --- | --- | --- | --- |
| Unfavorable growth conditions (S) | --- | --- | --- |  |  |  | -0.463* |  |  |
| Favorable growth conditions (S) | --- | --- | --- | --- | --- | --- | --- | --- | --- |
| Silty soil (S) |  |  |  | --- | --- | --- | --- | --- | --- |
| Carbonat bedrock/low soil clay content (S) | --- | --- | --- | --- | --- | --- | --- | --- | --- |
| High plant available water content (S) | --- | --- | --- | --- | --- | --- | --- | --- | --- |
| Soil depth (S) |  |  | 0.445** |  |  | 0.471** |  |  | 0.535*** |
| Interaction effects with corresponding other land-use types included | | | | | | | | | |
| n | 148 | | | 148 | | | 148 | | |
| R² | 0.748 | | | 0.621 | | | 0.649 | | |
| adj. R² | 0.606 | | | 0.500 | | | 0.563 | | |

Note: *** = p<0.01; ** = p<0.05; * = p<0.10; ^+^ = p<0.15; ‘---' = if variable was not used in the regression. Significances are based on bootstrapped standard errors.

**Appendix S12**

PCA results of all species correlated with rooting characteristics (all Pearson’s r > 0.4, p < 0.001), eigenvalues and component loadings are given; Components (C): 1= fresh to humid, nutrient-rich and sparse forest site; 2= fresh to humid field site; 3= south-alpine beech forest site; 4= nutrient-rich humid and wet grassland site; 5= nutrient-low, acid and dry lawn site; 6= nutrient-rich ruderal site; 7= soaked, humic-mouldy, moderately acid to acid forest site. Furthermore, in the last column, the coefficient of multiple correlation between components (C1-7) with all vegetation and site components/variables dependent on land-use types is provided.

| **Total variance explained** | | | | | | | | | **Coefficient of multiple correlation** | | |
| --- | --- | --- | --- | --- | --- | --- | --- | --- | --- | --- | --- |
| **Components** | **Eigenvalues** | | | | | | | |  |  |  |
|  | **Total** | | **% of variance** | | | **cumulative (%)** | | | **IM / UG / F** | | |
| C1 | 7.247 | | 24.989 | | | 24.989 | | | 0.890 / 0.864 / 0.957 | | |
| C2 | 4.281 | | 14.762 | | | 39.751 | | | 0.917 / 0.948 / 0.837 | | |
| C3 | 3.289 | | 11.343 | | | 51.094 | | | 0.942 / 0.903 / 0.927 | | |
| C4 | 2.702 | | 9.318 | | | 60.412 | | | 0.738 / 0.820 / 0.887 | | |
| C5 | 2.316 | | 7.986 | | | 68.398 | | | 0.868 / 0.898 / 0.906 | | |
| C6 | 1.392 | | 4.799 | | | 73.197 | | | 0.900 / 0.890 / 0.871 | | |
| C7 | 1.166 | | 4.022 | | | 77.218 | | | 0.907 / 0.955 / 0.903 | | |
| **Rotated component matrix** | | | | | | | | | | | |
|  | | **Components** | | | | | | | | | |
| **Plant species** | | C1 | | C2 | C3 | | C4 | C5 | | C6 | C7 |
| *Melica nutans* | | **0.991** | | -0.005 | 0.086 | | 0.016 | -0.012 | | -0.002 | -0.014 |
| *Euphorbia dulcis* | | **0.990** | | -0.004 | 0.048 | | 0.015 | -0.008 | | -0.002 | -0.009 |
| *Dryopteris filix-mas* | | **0.990** | | -0.004 | 0.048 | | 0.015 | -0.008 | | -0.002 | -0.009 |
| *Rubus idaeus* | | **0.983** | | -0.007 | 0.108 | | 0.017 | -0.015 | | -0.003 | -0.015 |
| *Daphne mezereum* | | **0.973** | | -0.006 | 0.137 | | 0.001 | -0.015 | | -0.003 | 0.013 |
| *Hepatica nobilis* | | **0.903** | | -0.012 | 0.246 | | 0.057 | -0.029 | | -0.004 | -0.053 |
| *Paris quadrifolia* | | **0.762** | | -0.016 | -0.012 | | 0.058 | -0.053 | | -0.004 | 0.022 |
| *Mentha arvensis* | | -0.010 | | **0.834** | -0.015 | | -0.023 | -0.053 | | 0.014 | -0.035 |
| *Zea mays* | | -0.005 | | **0.819** | -0.002 | | -0.019 | -0.014 | | 0.267 | 0.003 |
| *Rorippa sylvestris* | | -0.009 | | **0.811** | -0.011 | | -0.031 | -0.042 | | 0.124 | -0.017 |
| *Convolvulus arvensis* | | -0.012 | | **0.765** | -0.021 | | -0.027 | -0.070 | | 0.049 | -0.050 |
| *Setaria glauca* | | -0.009 | | **0.664** | -0.012 | | -0.028 | -0.045 | | **0.565** | -0.022 |
| *Fagus sylvatica* | | 0.004 | | -0.016 | **0.959** | | 0.013 | -0.042 | | -0.005 | -0.052 |
| *Luzula nivea* | | 0.176 | | -0.010 | **0.921** | | -0.013 | -0.033 | | -0.004 | -0.024 |
| *Melittis melissophyllum* | | **0.612** | | -0.013 | **0.780** | | 0.001 | -0.036 | | -0.005 | -0.034 |
| *Oxalis acetosella* | | 0.128 | | -0.026 | **0.769** | | 0.214 | -0.033 | | -0.008 | 0.067 |
| *Luzula sylvatica* | | 0.103 | | -0.028 | 0.050 | | **0.782** | 0.175 | | -0.011 | 0.020 |
| *Hypnum cupressiforme* | | -0.029 | | -0.005 | -0.040 | | **0.737** | -0.133 | | -0.004 | 0.177 |
| *Maianthemum bifolium* | | 0.112 | | -0.029 | 0.221 | | **0.713** | -0.023 | | -0.009 | 0.065 |
| *Phyteuma betonicifolium* | | -0.055 | | -0.039 | -0.002 | | **0.710** | 0.195 | | -0.012 | 0.182 |
| *Briza media* | | -0.015 | | -0.024 | -0.029 | | -0.004 | **0.872** | | -0.012 | -0.038 |
| *Lotus corniculatus agg.* | | -0.025 | | -0.066 | -0.047 | | -0.110 | **0.776** | | -0.020 | 0.050 |
| *Potentilla aurea* | | -0.026 | | -0.042 | -0.028 | | 0.091 | **0.728** | | -0.015 | -0.154 |
| *Potentilla erecta* | | -0.047 | | -0.069 | -0.011 | | 0.203 | **0.692** | | -0.021 | 0.233 |
| *Carex hirta* | | -0.006 | | 0.246 | -0.008 | | -0.014 | -0.029 | | **0.953** | -0.017 |
| *Digitaria ischaemum* | | -0.006 | | 0.246 | -0.008 | | -0.014 | -0.029 | | **0.953** | -0.017 |
| *Larix decidua* | | -0.026 | | -0.055 | 0.007 | | 0.094 | 0.008 | | -0.017 | **0.886** |
| *Avenella flexuosa* | | -0.027 | | -0.033 | -0.024 | | 0.311 | 0.090 | | -0.015 | **0.777** |
| *Picea abies* | | 0.032 | | -0.040 | -0.012 | | **0.570** | -0.133 | | -0.013 | **0.600** |

Extraction Method: Principal Component Analysis. Rotation Method: Varimax with Kaiser Normalization. Only factor loading values > 0.50 or < -0.50 are bold.

**Appendix S13: Technical Appendix**

**Methods for soil analysis**

A capillarimeter and a high-pressure membrane apparatus were used to determine the pore size distribution. Soil bulk density (*ρ****b***) was measured by drying of the ring samples at 105ºC to constant weight and dividing the oven-dried mass of the samples by the sample volume (Hartge 1978, Austrian norm L 1068). Soil particle density (*ρp*) was determined using the capillary pycnometer method with alcohol (Klute 1986, Austrian norm B 4413). To obtain the total soil porosity, soil bulk density was divided by soil particle density. The particle size distribution of the sand and part of the silt fraction (from 2000 µm to 40 µm) was determined by sieving (Klute 1986, Schlichting et al. 1995). The smaller fractions between 40 µm and 2 µm were analysed with a Centrifugal Particle Size Analyser (SA-CP 3, SHIMADZU Gmbh, Korneuburg/Vienna, Austria). Particle-size distribution was used to determine soil texture according to the textural classes of the Austrian norm L 1061. Soil organic C was obtained by wet digestion with K-dichromate (Walkley and Black method), and the organic matter was estimated using the Van Bemmelen factor. Soil pH measurements were conducted in a suspension of soil in distilled water and either in 0.01 M CaCl2-solution or in 1 M KCl, using a Beckman's pH meter in a soil:liquid suspension of 1:2.5.

**Statistical details**

For each rooting parameter (root mass, root length, rooting depth), a regression model was computed in order to investigate the hypothesized relationships illustrated in Figure 1. Data for these analyses consisted of 152 samples (nineteen of the 171 original samples were excluded because they could not be clearly assigned to a specific land-use type).

The impact of the components and variables was modelled allowing for differences in dependence of the land-use types:

$r_{i}={c+LU+x}_{i}^{'}\beta+y_{i}\gamma+\left( x_{i}^{'}\#LU \right)\beta_{LU}+\left( y_{i}^{'}\#LU \right)\gamma_{LU}+\varepsilon_{i}$,

where $x_{i}$ summarizes all site and $y_{i}$ all vegetation components/variables for sample $i$, $LU$ denotes the three different land-use types (forest, intensively used hay meadow, agriculturally unused grasslands) with the reference land-use type intensively used hay meadow excluded,$\left( x_{i}^{'}\#LU \right)$ indicates the interaction term of the site variables with the land-use type allowing for different impacts dependent on the corresponding land-use type, the same expression ($y_{i}^{'}\#LU$*)* for the vegetation components/variables is included, and $\varepsilon_{i}$ is the remainder noise. The corresponding dependent variable (i.e. root mass, root length or rooting depth) is denoted $r_{i}$ for sample *i*. The coefficients (coefficient vectors) to be estimated are $c, \beta, \gamma,\beta_{LU}$ and $\gamma_{LU}$, as well as $LU$, which summarises the coefficients modelling the different level effects in dependence of the considered land-use type (*c*, *LU*). Depending on the chosen reference land-use type, $\beta_{LU}$ displays the difference in the impact of other land-use types in comparison to the reference type (i.e., a statistically significant positive estimate denotes a larger impact than the impact of the reference land-use type).

The necessity of allowing for different level and slope effects was tested using an appropriate F-test with the null hypothesis that all these additional parameters, due to the different land-use types, are not statistically different from zero. Furthermore, the impact of the land-use types was investigated using the null hypothesis of no influence with respect to the slopes (i.e., $H_{0}:\beta_{LU}=0=\gamma_{LU}$).

Additionally, we counted how often the estimates of the components/variables were statistically different from each other across land-use types but within a model, i.e., to quantify the impact of the land-use types across the models. Within each model, the comparison of the standardised coefficients is appropriate to evaluate the size (and sign) of the association. Similarly, we counted how often a variable, within its category (*richness*, *community composition*, *cover of functional types*, *community-level traits*, or *site characteristics*), was significant across all models in order to quantify the importance of the categories for the rooting parameters.

The R-squared goodness-of-fit statistic is provided for each equation separately. For ease of interpretation and comparability of the impacts of the components/variables within a regression model, standardised regression coefficients were employed (i.e., a change in a variable is given in standard deviation units). The set of components and of independent variables was investigated for multicollinearity; for this purpose, the variance inflation factor (VIF) was computed. Each regression was checked for normality of the residuals (Shapiro-Wilk test), single influential observation units (Cook’s distance and relative change in the estimates) and evidence of heteroscedasticity (Breusch-Pagan test).

Although the number of variables was reduced using PCA, there were still many parameters in the model due to the interaction effects. Therefore, we used a two-step approach (Fig. 4). In the first step, just three land-use types with sufficient samples (forest, intensively used hay meadow, agriculturally unused grasslands) were included in the regression model, and the assumed relationships were estimated and statistically tested (i.e., the inductive step of the analyses). In the second step, only the components/variables with a p-value less than 0.15 in the first step were further employed in the regression model now with all land-use types with the reference land-use type arable land. Therefore, the relationships established for the remaining three land-use types (arable land, lightly used hay meadow, pasture) have a more explorative character, i.e. should not be considered as statistically tested yet.

All statistical analyses were conducted with Stata/MP 13.1 for Windows.

In order to identify similar sites according to their key species distribution we employed a PCA. For each of the resulting components an auxiliary linear regression with all vegetation and site components/variables as input was computed in order to get the coefficient of multiple correlation (R^2^).
